# Beighton Scoring System Use in Generalized Joint Hypermobility Studies Has Greater Scientific Rigor Than Joint‐Specific or Arthroscopy Joint Hypermobility Studies

**DOI:** 10.1002/ars2.70000

**Published:** 2026-05-04

**Authors:** John Nyland, Essa Gul, Jonathon Lewis, Justin Givens, Ryan Krupp, Jarod Richards

**Affiliations:** ^1^ Norton Orthopedic Institute Louisville Kentucky U.S.A.; ^2^ Department of Orthopaedic Surgery University of Louisville Louisville Kentucky U.S.A.

## Abstract

**Purpose:**

To compare the scientific rigor of Beighton Scoring System (BSS) use in generalized joint hypermobility (JH) studies (healthy subject injury risk/rate, physiological or kinesiological function determination) and joint‐specific or arthroscopy JH studies; to identify the most commonly used BSS score thresholds; and to describe ways to improve BSS score use for improved surgical and clinical decision‐making.

**Methods:**

Following the Preferred Reporting Items for Systematic Reviews and Meta‐analyses guidelines, the PubMed, EBSCO Host, and Web of Science databases were searched using “Beighton score” and “sports injury outcome” terms. Study purpose, publication year, female or male subject number, age and group type, measurement tools, BSS criteria, results, and conclusions data were extracted.

**Results:**

Twenty‐eight generalized JH studies (43.8%, 28/64) involving 12,138 subjects (6512 females) and 36 joint‐specific or arthroscopy JH studies (56.3%, 36/64) involving 7351 subjects (3441 females) were identified. Overall, most studies reported that BSS scores influenced most/all (54.7%, n = 35/64) or some (26.6%, 17/64) study outcomes and most were Evidence Level II (57.8%, n = 37/64) or III (35.9%, n = 23/64). Most generalized JH studies used a BSS score ≥4 (n = 13, 46.4%) or BSS ≥5 (n = 7, 25%) while most joint‐specific or arthroscopy JH studies used a BSS score ≥4 (n = 17, 47.2%), a BSS score ≥5 (n = 9, 25%), or the full BSS score scale (n = 7, 19.4%). Joint‐specific and arthroscopy JH studies were more recently published (2020.2 ± 3.5 vs 2014.2 ± 6.8, *P* < .001). Generalized JH studies more frequently reported separate subject sex and age data (53.6%, n = 15/28) while joint‐specific or arthroscopy JH studies more often combined this information (92.7%, 33/36) (*P* = .001). Most generalized JH studies were Evidence Level II (85.7%, 24/28) while most joint‐specific or arthroscopy JH studies were Evidence Level III (52.8%, 19/36) (*P* < .001). Group study quality and bias risk was comparable; however, generalized JH studies had more prospective research designs (96.4%, 27/28 vs 58.3%, 21/36 (*P* < .001).

**Conclusions:**

Generalized JH studies had more prospective research designs, had higher evidence levels, and more frequently reported separate subject age and sex details. Greater use of these characteristics in joint‐specific or arthroscopy JH studies may strengthen surgical and clinical decision‐making and patient outcome prediction validity.

**Level of Evidence:**

Level IV, narrative review of Level I to IV studies.

## Graphical abstract

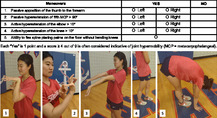

Joint hypermobility (JH) is the capacity for a joint to move beyond normal limits along physiological axes.^1^ While often benign and asymptomatic, JH has been increasingly associated with musculoskeletal injury and altered postoperative recovery, particularly among physically active individuals and patients undergoing arthroscopic surgery. Underlying JH can be attributed to pathophysiological collagen type imbalances, extracellular proteins, or hormonal factors within the extracellular matrix linked to hereditary connective tissue disorders.^2^, ^3^ Proprioceptive acuity may also be reduced with JH.^4^, ^5^ Knowing where athletes are on the JH spectrum may improve the efficacy of injury or reinjury prevention programs.^1^, ^6^

Sports‐active female individuals more commonly have JH, ranging from 10% to 15%,^7^ being more common in Asian and Black individuals than in White individuals.^8^ Patterns of JH are highly variable, related to sex, mechanical forces, lifestyle habits, occupation, and trauma, which are causally independent and may manifest at different ages.^1^, ^9^, ^10^, ^11^ While a specific hypermobile joint may exist in many generalized JH cases, these terms generally refer to separate clinical entities with differing etiologies. For example, knee JH may be associated with altered bony morphology, concomitant intra‐articular pathology, or genetic factors leading to a knee‐specific JH phenotype without generalized JH.^9^ Effective pre‐sports participation screenings for generalized JH may help with primary musculoskeletal injury prevention decisions about safe training practices such as avoiding loaded, end range of motion, and overhead weight training movements to prevent anterior glenohumeral joint (GHJ) instability. As JH may contribute to both increased primary a secondary joint injury risk, to optimize surgical and clinical decision‐making, both generalized and joint‐specific JH assessments should be performed.^1^, ^9^, ^12^

Designed by Dr. Peter H. Beighton, an orthopaedic surgeon and clinical geneticist with an epidemiology background, the Beighton Scoring System (BSS) is the most commonly used generalized JH assessment criteria (Figure 1).^1^, ^13^ Scores generated by the BSS are influenced by subject sex, age, ethnicity, strength and stretching training practices, warming up, and cooling down.^13^, ^14^, ^15^, ^16^ The BSS is a modification of the Carter and Wilkinson Scoring System,^17^, ^18^ decreasing its difficulty by changing from passive hyperextension of all fingers to passive extension of just the fifth finger metacarpophalangeal joint beyond 90°.^19^, ^20^, ^21^ In healthy college students, a BSS score threshold of ≥5 identified 36.7% (female) and 13.7% (male) JH rates,^22^ while a BSS score ≥4 identified JH in 51% of female students and 27.4% of male students.^23^ Using a BSS score threshold of ≥4, generalized JH rates of 66%, 24%, and 63% have been reported in female dance students,^24^ male rugby players,^25^ and female netball players,^26^ respectively, confirming sex and sport‐specific JH differences. The 2017 International Ehlers‐Danlos Syndrome Consortium recommended BSS score thresholds of ≥6 for prepubertal children and adolescents, ≥5 for pubertal men and women, and ≥4 for individuals aged ≥50 years.^13^

**FIGURE 1:**
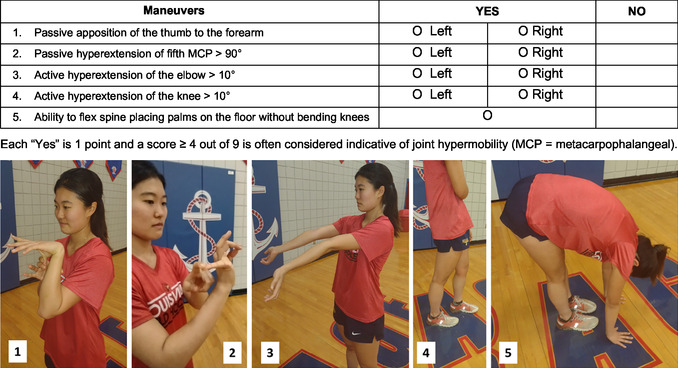
The Beighton Scoring System (BSS) criteria for generalized joint hypermobility.

Questions exist about BSS interpretation, optimal cut‐off thresholds,^27^ and its use for guiding surgical and clinical decisions.^2^, ^9^, ^13^ For example, the BSS component of increased genu recurvatum JH has been linked to both primary anterior cruciate ligament (ACL) injury and graft injury.^28^, ^29^ Similarly, shoulder or hip JH may predispose individuals to instability syndromes or poor surgical outcomes with increased pain and decreased function, emphasizing the added need for focused joint‐specific evaluations. To improve primary and secondary injury prevention strategies, information obtained from BSS scores should be integrated with focused joint‐specific clinical examination findings, individual patient characteristics, injury mechanisms, and patient/family medical histories. The purpose of this review was to compare the scientific rigor of BSS use in generalized JH studies (healthy subject injury risk/rate, physiological or kinesiological function determination) and joint‐specific or arthroscopy JH studies, to identify the most commonly used BSS score thresholds, and to describe ways to improve BSS score use for better surgical and clinical decision‐making. The hypothesis was that both study types would display comparable scientific rigor (evidence levels, study design, details of subject sex and age, study quality, and bias risk).

## METHODS

### Eligibility Criteria

In a narrative review format^30^ and in accordance with Preferred Reporting Items for Systematic Reviews and Meta‐analyses checklist,^31^ the PubMed, EBSCO Host, and Web of Science databases were searched using the following search terms: “Beighton score” and “sports injury outcome.” The following search term strategy was used: *(“Beighton”[All Fields] OR “Beighton s”[All Fields]) AND (“score”[All Fields] OR “score s”[All Fields] OR “scored”[All Fields] OR “scores”[All Fields] OR “scoring”[All Fields] OR “scorings”[All Fields]) AND (“injurie”[All Fields] OR “injuried”[All Fields] OR “injuries”[MeSH Subheading] OR “injuries”[All Fields] OR “wounds and injuries”[MeSH Terms] OR (“wounds”[All Fields] AND “injuries”[All Fields]) OR “wounds and injuries”[All Fields] OR “injurious”[All Fields] OR “injury s”[All Fields] OR “injured”[All Fields] OR “injuries”[All Fields] OR “injury”[All Fields]) AND (“outcome”[All Fields] OR “outcomes”[All Fields]).*

### Study Selection and Data Extraction

From this initial search, article titles and abstracts were reviewed by the primary investigator (J.N.). Only English‐language studies were included. Editorials, reviews, commentaries, meeting abstracts, dissertations, and theses were not included. No publication year limitations were applied. The primary investigator then reviewed each study to confirm that each used the BSS criteria and involved patients or healthy subjects representative of sport‐active individuals likely to potentially undergo arthroscopy. For each accepted study, the following information was extracted by the primary investigator and confirmed by a co‐investigator (R.K.): Publication year, Study purpose, Subjects/Methods, Tools, BSS Criteria, Results, and Conclusions (Supporting Information: Appendix I).

### Quality Assessment

Each study contributing to this review was evaluated using the Methodological Items for Non‐Randomized Studies (MINORS) assessment, with a score of “0” indicating that a particular item was not reported, a score of “1” indicating that it was reported but was inadequate, and a score of “2” indicating that an item was both reported and adequate. The maximum MINORS score for noncomparative studies is 16, and for comparative studies is 24.^32^ For randomized controlled trial studies, the revised Cochrane Risk‐of‐Bias tool for randomized studies (ROB2) was used.^33^ The primary investigator performed the initial review of potential studies for quality and relevance during the screening and data abstraction process. To better control for selection bias, however, co‐investigators (R.K. and J.L.) also reviewed each potential study to confirm whether it should be included. Where disagreements existed after study review, a fellowship‐trained orthopaedic shoulder surgeon (R.K.) made the final decision.

### Statistical Analysis

Contributing study source, design, evidence level, publication year, subject number, age and gender information, BSS criteria use, study quality, bias risk, and study outcome efficacy were statistically compared. For continuous variables, either unpaired t‐tests or one‐way analysis of variance tests with Tukey post hoc tests were used to delineate group differences. For categorical variables, chi‐square or Fisher's exact tests were used. An alpha level of *P* ≤ .05 was selected to indicate statistical significance. All statistical comparisons were performed with specialized software (SPSS version 29.0, IBM‐SPSS, Chicago, IL, USA).

## RESULTS

### Search Results

**FIGURE 2:**
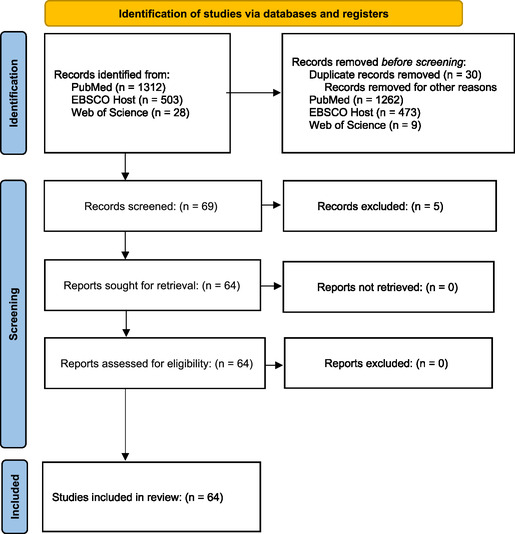
Record identification and exclusion PRISMA flowchart.

**FIGURE 3:**
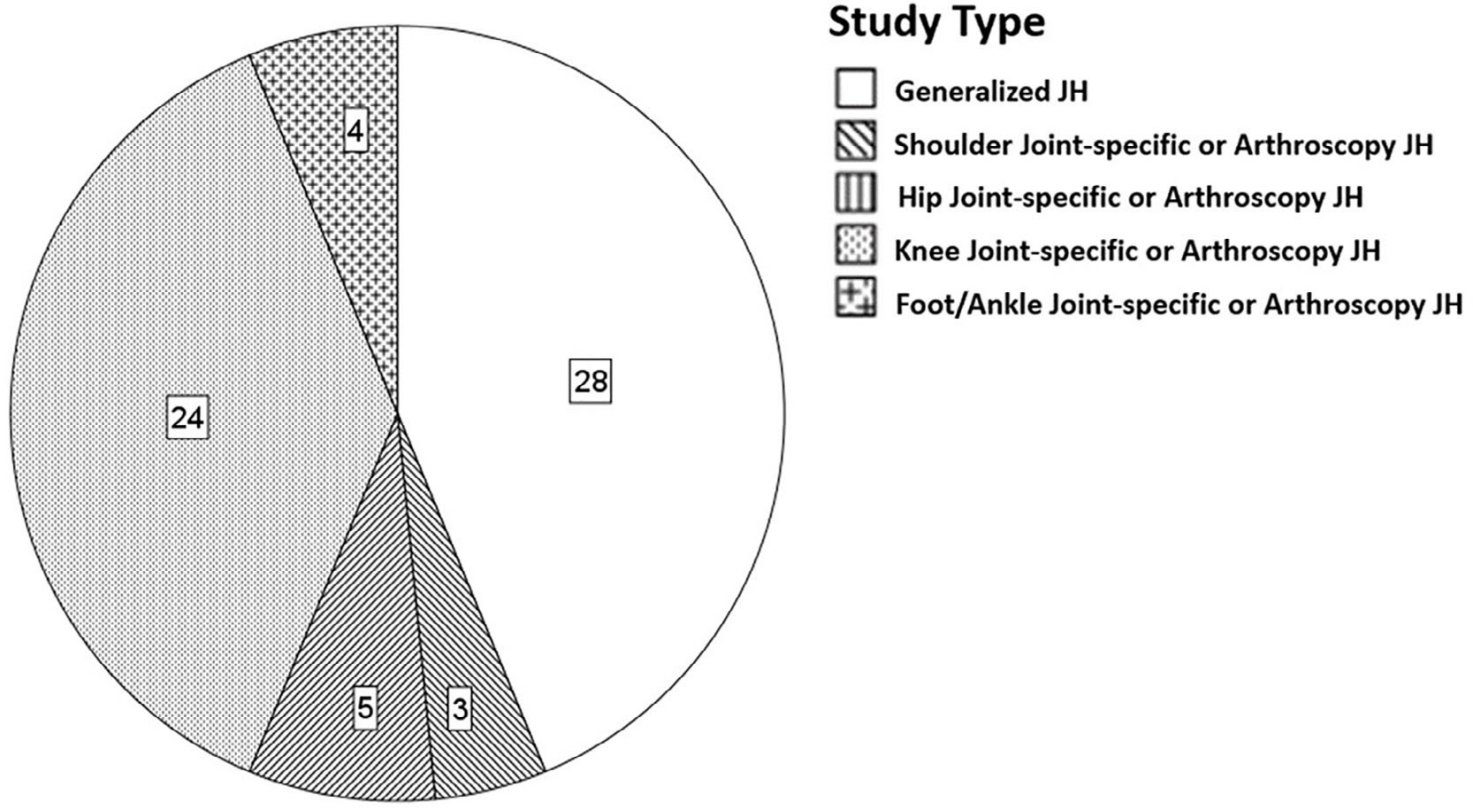
Pie chart of the generalized joint hypermobility and joint‐specific or arthroscopy joint hypermobility studies using the Beighton Scoring System score criteria that contributed to this review.

The initial search identified 1312, 503, and 28 records in the PubMed, EBSCO Host, and Web of Science databases, respectively. In addition to duplicate record removal, further title and abstract review led to the removal of an additional 1262, 473, and 9 records from the PubMed, EBSCO Host, and Web of Science databases, respectively. This created a total of 64 records that underwent full text review and were accepted for narrative review inclusion (Figures 2 and 3) (Table 1).^2^, ^9^, ^17^, ^22^, ^23^, ^24^, ^25^, ^34^, ^35^, ^36^, ^37^, ^38^, ^39^, ^40^, ^41^, ^42^, ^43^, ^44^, ^45^, ^46^, ^47^, ^48^, ^49^, ^50^, ^51^, ^52^, ^53^, ^54^, ^55^, ^56^, ^57^, ^58^, ^59^, ^60^, ^61^, ^62^, ^63^, ^64^, ^65^, ^66^, ^67^, ^68^, ^69^, ^70^, ^71^, ^72^, ^73^, ^74^, ^75^, ^76^, ^77^, ^78^, ^79^, ^80^, ^81^, ^82^, ^83^, ^84^, ^85^, ^86^, ^87^, ^88^, ^89^, ^90^

**TABLE 1:** Generalized JH and Joint‐Specific or Arthroscopy JH Study Source, Design, Evidence Level, Number of Female Subjects, Number of Male Subjects, Study Population, and Beighton Scoring System (BSS) Criteria

	Study	Design	Evidence Level	# Females	# Males	Age Females	Age Males		Population Group	BSS Score Criteria
Generalized JH	Armstrong R, Greig M.^34^	Prospective cross‐sectional	II	208	78	20.03 ± 0.03	20.6 ± 0.5		Rugby, netball, dance, and control subjects	≥4/9
	Bronner S, Bauer NG.^39^	Retrospective cohort	III	140	40	18.1 ± 0.53	18.28 ± 1.04		Modern dance program students	5‐9/9 high; 3‐4/9 medium; 0‐2/9 low
	Bukva B, et al.^40^	Retrospective cohort	III	7	17	13.86 ± 2.85	16.29 ± 4.88		Artistic gymnasts	≥5/9
	Clinch J, et al.^43^	Prospective cross‐sectional	II	3061	2961	13.8, *research clinic for 14 yr olds*			UK population‐based	≥4/9
	Collinge R, Simmonds JV.^2^	Prospective cohort	II	0	33	NA	24.4 ± 4.8		Professional soccer	≥4/9; ≥7/9 excessive
	Decoster LC, et al.^44^	Prospective cohort	II	114	150	15.5, range = 12‐19			Junior, senior high school adolescent athletes	≥5/9
	Frisch A, et al.^47^	Prospective cohort	II	0	67	15.0 ± 2, range = 13‐19			Youth soccer	≥4/9
	Hanzikova I, et al.^49^	Prospective cohort	II	17	25	22.5 ± 2.7		23.3 ± 1.3	Team sport participants	≥5/9 for women; ≥4/9 for men
	Hawke F, et al.^50^	Prospective cohort	II	20	10	10.7 ± 2.3			Healthy children	≥5/9
	Johnson A, et al.^54^	Prospective cohort	II	0	36	24.44 ± 4.95			Elite professional soccer	≥5/9
	Juul‐Kristensen B, et al.^56^	Prospective matched comparative	III	27	0	14.2 ± 0.3		NA	Healthy school children	≥6/9 and one JH knee
	Konopinski M, et al.^60^	Prospective cohort	II	0	80	NA		24.45 ± 4.6	Elite professional soccer	≥4/9
	Krivickas L, Feinberg J.^61^	Prospective cohort	II	70	131	19.6 ± 1.2		19.8 ± 1.5	College athletes	4‐6/9; 7‐9/9 extreme
	Nicolay RW, et al.^70^	Prospective cohort	II	0	73	NA		NR	College football	≥4/9
	Rejeb A, et al.^74^	Prospective cohort	II	0	226	NA		14.2 ± 1.7, range = 10‐18	Adolescent athletes	0‐9/9
	Russek L, & Errico D^23^	Prospective cohort	II	147	121	20.0 ± 1.68			Healthy college students	≥5/9
	Scheper M, et al.^24^	Prospective cohort	II	36	0	20.1 (range = 17‐27)		NA	Professional dancers	≥4/9
	Scheper M, et al.^22^	Prospective cross‐sectional cohort	II	72	0	19.6 ± 2.2		NA	Art, dance and theater students	≥4/9
	Schmidt H, et al.^76^	Prospective cohort	II	96	36	14.0 ± 0.9			Adolescent athletes	Compared ≥4/9, ≥5/9, ≥6/9
	Skwiot M, et al.^77^	Prospective cohort	II	58	19	20.03 ± 2.67			Jazz dancers	≥4/9
	Smith R, et al.^78^	Prospective cohort	II	200	0	11 ± 2.5		NA	Junior netball players	0‐2/9 not JH; 3‐4 moderate; 5‐9 distinct
	Soper K, et al.^26^	Prospective cohort	II	27	0	19.3 ± 3.7; range = 14‐26		NA	Elite netball players	≥4/9; 4‐6/9 high; 7‐9/9 distinct
	Stewart D, Burden S.^25^	Prospective cohort	II	51	0	23.6 ± 3.3		NA	First division club rugby players	4‐6/9; 7‐9/9 extreme
	Sueyoshi T, et al.^79^	Prospective case‐control	III	47	0	15.83 ± 0.87		NA	High school volleyball players	0‐9/9
	Tobias JH, et al.^82^	Prospective cohort	II	1634	1267	JH at mean 13.8, MS pain at 17.8			UK population‐based	≥6/9
	Van Meulenbroek et al.^84^	Prospective cross‐sectional cohort	II	47	15	17.0 ± 3.8			Healthy adolescents	≥6, <18 yrs of age; ≥5, ≥18 yrs of age
	van Rijn et al.^85^	Prospective cohort	II	185	0	19.1 ± 1.3		NA	Preprofessional contemporary dancers	0‐9/9
	Zhong G, et al.^89^	Prospective cohort	II	299	190	20.7 ± 4.6			Healthy college students	≥4/9
Shoulder Joint‐Specific or Arthroscopy JH	Cameron K, et al.^41^	Prospective cross‐sectional cohort	II	84	630	18.7 ± 0.9		18.8 ± 1.0	Healthy college students	≥2/9 (95^th^ percentile)
	Khan U, et al.^58^	Retrospective cohort	III	4	12	29.9 ± 8.9, range = 17‐50			Patients s/p failed Latarjet surgery	0‐9/9
	Lim J, et al.^63^	Retrospective cohort	III	6	75	23.8 ± 4.6			Patients s/p Bankart repair	≥4/9
Hip Joint‐Specific or Arthroscopy JH	Chandrasekaran S, et al.^42^	Retrospective case series	IV	87	15	16.2, range = 13.2‐17.97		16.7, range = 15.0‐17.95	Adolescent patients with hip labral tears	≥4/9
	Firat A, et al.^46^	Retrospective cohort	III	53	37	37.9 ± 9.8			Patients s/p hip arthroscopy with extended interportal capsulotomy	0‐9/9
	Maldonado D, et al.^66^	Prospective matched comparative	III	127	18	34.9 ± 13.2			Patients s/p hip arthroscopy for labral tears and FAI compared to controls	≥4/9
	Mojica E, et al.^68^	Retrospective cohort	III	65	15	34.4 ± 10.2			Patients with iliopsoas tendinitis s/p hip arthroscopy for FAI compared to controls	≥4/9
	Naal F, et al.^69^	Retrospective cohort	III	118	114	34.9 ± 4.0			Patients with FAI	≥4/9
Knee Joint‐Specific or Arthroscopy JH	Astur D, et al.^35^	Prospective case series	IV	43	199	32.9 ± 11.88, range = 10‐72			Patients s/p ACLR, partial meniscectomy or both	≥4/9
	Batty L, et al.^37^	Retrospective cohort	III	319	299	18.9 ± 3.2			Patients with AL knee instability factors	≥4/9; GR > 10°
	Brinkman J, et al.^38^	Retrospective cohort	III	93	66	18.7 ± 3.2			Patients s/p ACLR using HTA + LET, soft tissue QA, or HTA.	≥4/9
	Feller J, et al.^45^	Retrospective case series	IV	4	21	18.5, range = 13.8‐28.7			Patients s/p ACLR + modified Ellison procedure	≥4/9
	Getgood A, et al.^48^	Randomized controlled	I	321	297	18.9, range = 14‐25			Patients s/p HTA ACLR with or without LET	≥4/9, or GR > 10°
	Helito C, et al.^51^	Prospective case‐control	III	49	41	28.5 ± 8.6			Patients s/p ACLR or ACLR + ALL compared to controls	≥5/9
	Hosseinzadeh N, et al.^52^	Retrospective case‐control	III	7	73	29.44 ± 8.06			Patients s/p ACLR w Quadrupled HTA with or without JH	≥4/8
	Juul‐Kristensen B, et al.^55^	Prospective matched comparative	II	38	37	Children 10.2 Adults 40.3			Patients with or without JH evaluated for knee function during static balance tasks	≥5/9 for children; ≥4/9 for adults
	Keizer M, et al.^57^	Prospective cohort	II	15	25	26.3, range = 18‐42			Coper and noncoper patients s/p ACLR evaluated for muscle activation during SLHD	0‐9/9
	Kim S, et al.^59^	Retrospective cohort	III	54	183	29.7 ± 9.8			Outcomes of patients with or without JH were evaluated 2‐ and 5‐years s/p ACLR	≥4/9
	Larson C, et al.^62^	Prospective case‐control	III	102	81	26.9, range = 12‐62			Outcomes of patients with or without JH and contralateral GR ≥ 2‐years s/p ACLR were evaluated	3 of 4 “+” criteria
	Lindskog J, et al.^64^	Prospective cohort	II	364	301	28.5 ± 8.6			Outcomes, RTS, and RTP of patients up to 2 yrs s/p ACLR were evaluated based on JH or not	≥5/9
	Lodhia, P. et al.^65^	Prospective cohort	II	113	115	18.9 ± 3.2			Outcomes ≥2 yrs s/p 4S or 5S HTA ACLR alone or + LET were evaluated for JH influence	0‐9/9
	Marmura H, et al.^67^	Retrospective cross‐sectional	III	318	297	19 ± 3			Evaluated KOOS validity for young, active patients with ACL tears	≥4/9 or GR > 10°
	Parmar R, et al.^71^	Retrospective comparative	III	133	0	18 ± 2.8			Patient outcomes ≥2 yrs s/p ACLR with QA, BPTB A, or HTA, with or without LET	≥4/9
	Pfeiffer TR, et al.^72^	Prospective observational	II	50	48	20 ± 5		20 ± 8	Determined if factors such as gender were associated with increased rotatory knee laxity in college athletes with no injury history	≥5/9
	Sahin S, et al.^75^	Prospective cross‐sectional	II	25	0	14.5 ± 1.8, range = 10.5‐17.2			Compared the influence of BSS score on volleyball player biomechanical risk factors during DLVJ, SLS, SLDL	0‐9/9
	Sundemo D, et al.^80^	Prospective cohort	II	40	56	24.6 ± 9.1			Compared rotatory knee laxity in patients ACL injured and contralateral knees based on JH	0‐4/9 low; 5‐9/9 high
	Sundemo D, et al.^81^	Prospective cohort	II	210	146	25.9 ± 8.7			Evaluated 1‐year s/p ACLR for effects of JH on RTS, PROM, hop tests, strength and reinjury	≥5/9
	Vaishya R, Hasija R.^83^	Retrospective cohort	III	110	190	24.6 ± 0.9			Compared influence of JH prevalence in patients with and without ACL injury	≥4/9
	Westin M, et al.^87^	Prospective cohort	II	26	5	16.1 ± 0.5			Evaluated clinical exam and performance differences in adolescent alpine skiers s/p ACLR	≥5/9
	Zhang Z, et al.^88^	Retrospective comparative	III	115	189	31.4 ± 9.3, range = 14‐58			Evaluated the relationship between PTS and ATS in patients with an injured and with an intact ACL.	0‐9/9
	Ziegler C, et al.^90^	Retrospective cross‐sectional comparative	III	102	97	31.8, range = 21‐45			Compared patients ≥1 yr s/p revision ACLR with patients who underwent primary ACLR based on JH	0‐9/9
	Zsidai B, et al.^9^	Prospective observational	II	120	105	23.7 ± 1.9			Determined the 12‐month second ACL injury risk in patients with JH who RTS after ACLR	≥5/9
Ankle Joint‐Specific or Arthroscopy JH	Baillie P, et al.^36^	Retrospective cross‐sectional, case control	III	6	14	29 ± 4			Compared clinical exam findings between dancers with and without PAIS	≥5/9
	Hou Z, et al.^53^	Prospective cohort	II	20	15	29.6 ± 3.3			Compared the efficacy of balance training of patients with CAI with or without JH	≥4/9
	Porter M, et al.^73^	Prospective cohort	II	58	68	26.87 ± 8.47			Evaluated clinical outcomes for patients with CAI post‐MBG or LARS with respect to JH and bodyweight	≥5/9
	Wang A, et al.^86^	Retrospective cohort	III	42	26	29.7 ± 9.6			Compared RTS and clinical outcomes in patients with or without JH post‐MBG procedure or anatomic reconstruction	≥4/9

ALL, anterolateral ligament; ACLR, anterior cruciate ligament reconstruction; ATS, anterior tibial subluxation; BPTB A, bone‐patellar tendon‐bone autograft; BSS, Beighton Scoring System; CAI, chronic ankle instability; DLVJ, drop landing vertical jump; FAI, femoroacetabular impingement; GR, genu recurvatum; JH, joint hypermobility; HTA, hamstring tendon autograft; LARS, ligament augmentation reconstruction system; LET, lateral extra‐articular tenodesis; MBG, modified Brostrom‐Gould; MS, musculoskeletal; NA, not applicable; NR, not reported; PAIS, posterior ankle impingement syndrome; PTS, posterior tibial subluxation; PROM, patient reported outcome measurement(s); RTS, return to sports; RTP, return to performance; s/p, status post; SLHD, single leg hop distance; SLS, single leg squat; SLDL, single leg drop landing; 4S, 4 strand; 5S, 5 strand.

### Study Outcome Influence and Evidence Levels

Most studies observed that BSS scores influenced most/all (54.7%, n = 35/64) or at least some (26.6%, 17/64) primary study outcomes. Most studies were Evidence Level II (57.8%, n = 37/64) or Evidence Level III (35.9%, n = 23/64) studies. One Evidence Level I study was included (1.5%, n = 1/64), and 3 were Evidence Level IV (4.7%, n = 3/64) studies.

### Generalized JH and Joint‐Specific or Arthroscopy JH Study BSS Criteria Use Comparisons

Twenty‐eight generalized JH studies (43.8%, 28/64) of sport active populations were identified (n = 12,138, 6512 females). Of the 28 studies evaluating BSS criteria score influence on sport active populations, most used a BSS score ≥4 (n = 13, 46.4%) or BSS ≥ 5 (n = 7, 25.0%). Three studies used the full BSS scale (0‐9) (10.7%, 3/28). Two studies used a BSS score ≥6 (7.1%, 2/28). One study used a BSS score ≥5 for women and ≥4 for men. One study used a BSS score between 4 and 6. One study used a BSS score ≥6 for subjects aged <18 years and ≥5 for subjects aged ≥18 years. Of the 36 joint‐specific or arthroscopy JH studies (n = 7351, 3441 females) reporting BSS scores, 47.2% (17/36) used a BSS score ≥4, 25% (9/36) used a BSS score ≥5, 19.4% (7/36) used the full 0‐9 BSS scale, 1 study (2.8%, 1/36) used a BSS score = 3 or 4, 1 study used a BSS score ≥2, and 1 study used a BSS score ≥4 for adults and ≥5 for children. Three knee joint studies added the presence of ≥10° genu recurvatum JH at 1 knee to the BSS score cut‐off threshold criteria.

### Generalized JH and Joint‐Specific or Arthroscopy JH Study Publication Year and Level of Evidence Comparisons

Joint‐specific or arthroscopy JH studies were published more recently than generalized JH studies (2020.4 ± 3.5 vs 2014.4 ± 6.8, *P* < .001). However, generalized JH studies included more Level of Evidence Level II study designs (85.7%, 24/28) compared with joint‐specific and arthroscopy JH studies, which had mostly Level III study designs (52.8%, 19/36) (*P* < .001). Additionally, generalized JH studies more often reported separate female and male subject numbers and respective ages (57.1%, n = 16/28) compared with joint‐specific and arthroscopy JH studies, which more frequently combined sex and age data (92.7%, 33/36) (*P* = .001) (Table 2).

**TABLE 2:** Generalized JH and Joint‐Specific or Arthroscopy JH Study Subject Sex and Age Data Reporting Methods With Cited References

	Generalized JH Studies	Joint‐Specific or Arthroscopy JH Studies
Separate Subject Sex and Age Data	n = 16^22^, ^24^, ^25^, ^26^, ^34^, ^39^, ^40^, ^49^, ^56^, ^60^, ^61^, ^70^, ^74^, ^78^, ^79^, ^85^	n = 3^41^, ^42^, ^72^
Combined Subject Sex and Age Data	n = 12^2^, ^23^, ^43^, ^44^, ^47^, ^50^, ^54^, ^76^, ^77^, ^82^, ^84^, ^89^	n = 33^9^, ^35^, ^36^, ^37^, ^38^, ^45^, ^46^, ^48^, ^51^, ^52^, ^53^, ^55^, ^58^, ^59^, ^62^, ^63^, ^64^, ^65^, ^66^, ^67^, ^68^, ^69^, ^71^, ^72^, ^73^, ^75^, ^80^, ^81^, ^83^, ^86^, ^87^, ^88^, ^90^
Total	n = 28	n = 36

JH, joint hypermobility.

### Generalized JH and Joint‐Specific or Arthroscopy JH Study Efficacy and Quality Comparisons

Most studies observed that BSS scores influenced most/all (54.7%, n = 35/64) or some (26.6%, 17/64) study outcomes, with similar frequency in both study types (*P* = .32) (Table 3). Contributing studies displayed good‐to‐excellent bias control based on MINORS scores^32^ or the revised ROB2^33^ (Tables 4 and 5). The joint‐specific or arthroscopy JH study group had more comparative studies (31/36 (86%) vs. 18/28 (64%), while the generalized JH group had more noncomparative studies (10/28 (36%) vs 5/36 (14%), *P* = .04). Comparative joint‐specific or arthroscopy JH study MINORS scores (12.4 ± 2) were comparable to generalized JH study scores (13.5 ± 1, *P* = .33), suggesting moderate study quality and bias risk in both groups. Noncomparative generalized JH studies had slightly higher MINORS scores (22.2 ± 1) compared with noncomparative joint‐specific or arthroscopy JH studies (21.4 ± 2, *P* = .048); however, both group scores suggested moderate study quality and bias risk. A randomized controlled arthroscopy JH study^48^ was evaluated using both MINORS score criteria and the ROB2, with each suggesting high study quality and low bias risk.

**TABLE 3:** Generalized JH and Joint‐Specific or Arthroscopy JH Study Reported BSS Score Influence on Primary Study Outcomes With Cited References

BSS Score Influence on Primary Study Outcomes	Generalized JH Studies	Joint‐Specific or Arthroscopy Studies
Most/All	14 (50%)^2^, ^22^, ^24^, ^34^, ^39^, ^50^, ^54^, ^56^, ^60^, ^74^, ^78^, ^79^, ^82^, ^89^	21 (58.3%)^9^, ^36^, ^38^, ^41^, ^45^, ^48^, ^51^, ^53^, ^55^, ^58^, ^59^, ^62^, ^64^, ^68^, ^71^, ^75^, ^80^, ^83^, ^86^, ^88^, ^90^
Some	10 (35.7%)^23^, ^25^, ^26^, ^43^, ^44^, ^49^, ^61^, ^76^, ^77^, ^84^	7 (19.4%)^42^, ^52^, ^57^, ^63^, ^66^, ^67^, ^81^
None	4 (14.3%)^40^, ^47^, ^70^, ^85^	8 (22.2%)^35^, ^36^, ^65^, ^69^, ^72^, ^73^, ^87^
Total	28	36

BSS, Beighton Scoring System; JH, joint hypermobility.

**TABLE 4:** Methodological Index for Non‐Randomized Studies (MINORS) Scores

		Noncomparative Studies												
		Comparative Studies												
Type	Study	Clearly Stated Aim	Inclusion of Consecutive Samples	Prospective Data Collection	Endpoints Appropriate to Study Aim	Unbiased Study Endpoint Assessment	Assessment Tests Appropriate With Aim	<5% Sample Loss	*Prospective Study Size Calculation*	*Adequate Control Group*	*Contemporary Groups*	*Baseline Group Equivalence*	*Adequate Statistical Analysis*	Results
Generalized JH Studies	Armstrong R & Greig M.^34^	2	2	2	2	2	2	2	0	2	2	2	2	22
	Bronner S & Bauer NG.^39^	2	2	2	2	2	2	2	2	1	2	1	2	22
	Bukva B, et al.^40^	2	2	0	2	2	2	2	0					12
	Clinch J, et al.^43^	2	2	2	2	2	2	2	0	2	2	2	2	22
	Collinge R & Simmonds JV^2^	2	2	2	2	2	2	2	0					14
	Decoster LC, et al.^44^	2	2	2	2	2	2	2	0	2	2	2	2	22
	Frisch A, et al.^47^	2	2	2	2	2	2	0	0					12
	Hanzikova I, et al.^49^	2	2	2	2	2	2	2	2	2	2	2	2	24
	Hawke F, et al.^50^	2	2	2	2	2	2	2	0					14
	Johnson A, et al.^54^	2	2	2	2	2	2	2	0					14
	Juul‐Kristensen B, et al.^56^	2	2	2	2	2	2	2	0	2	2	2	2	22
	Konopinski M, et al.^60^	2	2	2	2	2	2	2	0	2	2	2	2	22
	Krivickas L & Feinberg J^61^	2	2	2	2	2	2	2	0					14
	Nicolay RW, et al.^70^	2	2	2	2	2	2	2	2	2	2	2	2	24
	Rejeb A, et al.^74^	2	2	2	2	2	2	2	0	2	2	1	2	21
	Russek L & Errico D^23^	2	2	2	2	2	2	2	2	2	2	2	2	24
	Scheper M, et al.^24^	2	2	2	2	2	2	2	0	2	2	2	2	22
	Scheper M, et al.^22^	2	2	2	2	1	2	2	0	2	2	2	2	21
	Schmidt H, et al.^76^	2	2	2	2	2	2	2	1	2	2	2	2	23
	Skwiot M, et al.^77^	2	2	2	2	2	2	2	0	2	2	2	2	22
	Smith R, et al.^78^	2	1	2	2	2	2	2	0					13
	Soper K, et al.^17^	2	2	2	2	2	2	2	0					14
	Stewart D & Burden S^25^	2	2	2	2	2	2	2	0					14
	Sueyoshi T, et al.^79^	2	2	2	2	2	2	2	0	2	2	2	2	22
	Tobias JH, et al.^82^	2	2	2	2	2	2	2	0	2	2	2	2	22
	Van Meulenbroek et al.^84^	2	2	2	2	2	2	2	0	2	2	1	2	21
	van Rijn R & Stubbe J^85^	2	2	2	2	2	2	2	0					14
	Zhong G, et al.^89^	2	2	2	2	2	2	2	0	2	2	1	2	21
Shoulder Joint‐Specific or Arthroscopy JH Studies	Cameron K, et al.^41^	2	2	2	2	2	2	2	0	2	2	2	2	22
	Khan U, et al.^58^	2	2	2	2	2	2	2	0					14
	Lim J, et al.^63^	2	2	0	2	2	2	2	0	2	2	2	2	20
Hip Joint‐Specific or Arthroscopy JH Studies	Chandrasekaran S, et al.^42^	2	2	0	2	2	2	0	0	1	2	1	2	16
	Firat A, et al.^46^	2	2	0	2	2	2	0	0					10
	Maldonado D, et al.^66^	2	2	2	2	2	2	2	0	2	2	2	2	22
	Mojica E, et al.^68^	2	2	0	2	2	2	2	2	0	2	2	2	20
	Naal F, et al.^69^	2	2	0	2	2	2	2	0	2	2	2	2	20
Knee Joint‐Specific or Arthroscopy JH Studies	Astur D, et al.^35^	2	2	2	2	2	2	2	0	2	2	2	2	22
	Batty L, et al.^37^	2	2	2	2	2	2	2	0	2	2	2	2	22
	Brinkman J, et al.^38^	2	2	0	2	2	2	2	0	2	2	2	2	20
	Feller J, et al.^45^	2	2	2	2	2	2	2	0					14
	Getgood A, et al.^48^	2	2	2	2	2	2	2	0	2	2	2	2	24
	Helito C, et al.^51^	2	2	0	2	2	2	2	0	2	2	2	2	20
	Hosseinzadeh N, et al.^52^	2	2	0	2	2	2	2	2	2	2	2	2	22
	Juul‐Kristensen B, et al.^55^	2	2	2	2	2	2	2	0	2	2	2	2	22
	Keizer M, et al.^57^	2	2	2	2	2	2	2	0	2	2	2	2	22
	Kim S, et al.^59^	2	2	0	2	2	2	0	0	2	2	2	2	18
	Larson C, et al.^62^	2	2	2	2	2	2	2	2	2	2	2	2	24
	Lindskog J, et al.^64^	2	2	2	2	2	2	2	0	2	2	2	2	22
	Lodhia, P. et al.^65^	2	2	2	2	2	2	2	0	2	2	2	2	22
	Marmura H, et al.^67^	2	2	0	2	2	2	0	0					10
	Parmar R, et al.^71^	2	2	0	2	2	2	2	0	2	2	2	2	20
	Pfeiffer TR, et al.^72^	2	2	2	2	2	2	2	0	2	2	2	2	22
	Sahin S, et al.^75^	2	2	2	2	2	2	2	0					14
	Sundemo D, et al.^80^	2	2	2	2	2	2	0	0	2	2	2	2	20
	Sundemo D, et al.^81^	2	2	2	2	2	2	2	2	2	2	2	2	24
	Vaishya R & Hasija R.^83^	2	2	0	2	2	2	2	0	2	2	2	2	20
	Westin M, et al.^87^	2	2	2	2	2	2	2	0	2	2	2	2	22
	Zhang Z, et al.^88^	2	2	0	2	2	2	2	0	2	2	2	2	20
	Ziegler C, et al.^90^	2	2	0	2	2	2	2	0	2	2	2	2	20
	Zsidai B, et al.^9^	2	2	2	2	2	2	2	0	2	2	2	2	22
Ankle Joint‐Specific or Arthroscopy JH Studies	Baillie P, et al.^36^	2	2	2	2	2	2	2	0	2	2	2	2	22
	Hou Z, et al.^53^	2	2	2	2	2	2	2	2	2	2	2	2	24
	Porter M, et al.^73^	2	2	2	2	2	2	2	2	2	2	2	2	24
	Wang A, et al.^86^	2	2	0	2	2	2	2	2	2	2	2	2	22

JH, joint hypermobility.

**TABLE 5:** Revised Cochrane Risk‐of‐Bias Tool for Randomized Studies (RoB2)^33^

	Random Sequence Generation	Allocation Concealment	Blinding of Participants and Personnel	Blinding of Outcome Assessment	Incomplete Outcome Data	Selective Reporting	Other Bias
Getgood AM, et al.^48^	+	+	+	+	+	+	+

*Note:* Results for Getgood A, et al.^48^ “+” = Low Risk of Bias; “‐” = High Risk of Bias; “?” = Unclear Risk of Bias. Associated Studies That Used Subsets of Data From This Study Were Performed by Batty et al.;^37^ Lodhia et al.;^65^ and Marmura et al.^67^

#### Generalized JH Study Results

Given the high number of generalized JH studies, the publication source, study purpose, results, and conclusions are displayed separately in Table S1. In summary, the JH characteristic as identified by the BSS score was useful for evaluating injury prevention strategies,^34^, ^39^ sex differences,^43^ delayed return to sports (RTS) timing,^2^ injury risk,^23^, ^26^, ^44^, ^60^, ^74^, ^76^, ^77^, ^78^, ^79^, ^82^, ^85^ running directional change kinematics,^49^ foot or lower limb alignment,^50^, ^54^ neuromuscular activation level changes,^56^ musculoskeletal and psychological complaints,^24^ impaired functional movement control,^17^ increased knee extensor torque,^84^ and anterior tibial translation.^89^ In summary, JH as determined by BSS scores influenced most/all,^2^, ^22^, ^24^, ^34^, ^39^, ^50^, ^54^, ^56^, ^60^, ^71^, ^78^, ^79^, ^82^, ^89^ some,^23^, ^25^, ^26^, ^43^, ^49^, ^61^, ^76^, ^77^, ^84^ or none^40^, ^47^, ^70^, ^85^ of the generalized JH study outcomes.

#### Shoulder Joint‐Specific or Arthroscopy JH Study Results

The following is a synopsis of shoulder joint–specific or arthroscopy JH studies. Cameron et al.^41^ studied the relationship between gender, JH (BSS score ≥ 4), and a history of GHJ instability in physically active college freshmen. Most participants (78%), however, had no signs of JH as only 11 subjects (1.5%) had BSS scores ≥4. Logistic regression revealed a relationship between JH and GHJ instability history (*P* = .023). When sex and race were controlled for, subjects with a BSS score ≥2 were nearly 2.5X more likely (odds ratio [OR] = 2.48, 95% confidence interval [CI] = 1.19, 5.20, *P* = .016) to have GHJ instability. A relationship was observed between sex and nearly all individual BSS score items. Although women had higher total BSS scores than men, sex (*P* = .658) and race (*P* = .410) were not related to GHJ instability history when other model variables were controlled for. Khan et al.^58^ evaluated the influence of BSS scores on patient outcomes post‐Perfailed Latarjet procedures. Sixteen patients (12 male, 4 female) underwent recurrent GHJ instability revision surgery. Of these patients, 9 were professional and 2 were amateur athletes. The mean age at revision was 29.9 ± 8.9 years (range = 17‐50 years). Revision indications were anterior GHJ instability (11 patients), posterior GHJ instability (4 patients), and both anterior and posterior GHJ instability (1 patient). Of the anterior GHJ instability cases, 54.5% were associated with coracoid process nonunion and 36.4% were associated with capsular failure (retear). All posterior instability cases had posterior capsulolabral injuries, and the mean BSS score in this group was ≥6/9. One patient had a coracoid process fracture nonunion and a posterior labral tear. They concluded that coracoid process fracture nonunion was the most common cause of failed Latarjet stabilization. Patients who returned with posterior GHJ instability had a high JH incidence but were successfully treated arthroscopically. Lim et al.^63^ evaluated GHJ bipolar bone defect characteristics in the presence of JH (BSS score ≥4) and clinical outcomes based on the “on‐track/off‐track” theory. They reported no glenoid bone defect group differences (JH group = 14.1%, non‐JH group = 14.4%). Off‐track lesions were identified in 39.4% (13/33) of the JH group and 14.6% (7/48) of the non‐JH group (*P* = .011). The mean Hill‐Sachs interval to glenoid track ratio was 83.1% in the JH group and 75.2% in the non‐JH group (*P* = .021). Additional remplissage procedures were more often performed in the JH group (48.5%; 16/33) than in the non‐JH group (16.7%; 8/48) (*P* = .002). Significant differences, however, were not observed for shoulder function scores and group injury recurrence rates. They concluded that patients with anterior GHJ instability and JH had wider Hill‐Sachs lesions and more off‐track lesions than did patients with normal GHJ laxity despite the lack of significant glenoid bone defect differences. The Hill‐Sachs lesion differences that were observed were not related to group functional outcome differences. In summary, JH as assessed by BSS scores influenced most/all^41^, ^58^ or some^63^ of the shoulder joint–specific or arthroscopy JH study outcomes.

#### Hip Joint–Specific or Arthroscopy JH Study Results

The following is a synopsis of hip joint–specific or arthroscopy JH studies. Chandrasekara et al.^42^ used a BSS score threshold of ≥4/9 to indicate JH among patients who had undergone hip arthroscopy for labral tears. Women had greater hip external rotation in hip flexion and greater femoral anteversion than men. To better address soft‐tissue laxity and impingement, women were also more likely to undergo capsular plication and iliopsoas fractional lengthening than men (88.3% vs 46.2%, and 77.9% vs 38.5%, respectively). Women had lower pre‐ and postoperative PROM scores. Hip labral injury patterns were different in men and women, dictating the selected arthroscopic approach. Using a standardized extended interportal capsulotomy, Firat et al.^46^ evaluated the influence of 0‐9/9 BSS score differences on visual analog scale (VAS) pain, Hip Disability and Osteoarthritis Outcome Score, and Modified Harris Hip Score (mHHS) among 90 patients (58.9% male) at 37.9 ± 9.8 years of age after hip arthroscopy. At ≥2 years' mean follow‐up (FU) (39.2 months), patients had improved clinical scores and a low revision rate. They concluded that this surgical approach was a safe procedure with satisfactory mid‐term results and high overall patient satisfaction. All study patients, however, had relatively low BSS scores. Maldonado et al.^66^ evaluated the influence of JH (BSS score ≥4) on patient outcomes after hip arthroscopy for symptomatic labral tears and femoral acetabular impingement (FAI) compared with a control group without JH (BSS score < 4). They reported no group differences for VAS pain, mHHS, Non‐Arthritic Hip Score, Hip Outcome Score‐Sports Specific Scale, and International Hip Outcome Tool‐12 (iHOT‐12) scores. The only group difference was that the JH group required more capsular plications. They concluded that for symptomatic FAI and labral tears, patients with JH may expect favorable outcomes with appropriate arthroscopic labral and capsular management at ≥2‐year FU. Comparable results were observed for the mHHS, Non‐Arthritic Hip Score, HOS‐SSS, and VAS pain scores, and patient acceptable symptomatic state and/or minimal clinically important difference mHHS, HOS‐SSS, and International Hip Outcome Tool‐12 frequencies of a pair‐matched control group without JH.

In a study to determine the influence of JH (BSS score high ≥4/9 vs low <4/9) on iliopsoas tendinitis in postoperative hip arthroscopy patients treated for FAI, Mojica et al.^68^ reported that increased JH was associated with greater iliopsoas tendinitis risk. For each 1‐point BSS score increase, there was 1.69x (95% CI, 1.25‐2.29; *P* < .001) increased postsurgical odds of iliopsoas tendinitis development. A high (≥4/9) BSS score was associated with increased iliopsoas tendinitis development likelihood (OR = 9.82; 95% CI, 2.79‐34.58; *P* < .001). In a study of the influence of JH on the outcomes of patients with FAI, Naal et al.^69^ reported that although there was a weak inverse relationship (r = −0.16 to −0.30) between high BSS score (≥4) and preoperative PROM values, significant relationships were not observed between BSS scores and postoperative PROM values or subjective failure rates. Patients who objectively failed, however, had lower BSS scores (<4) than did nonfailures (1.6 vs. 2.6; *P* = .049). They concluded that JH as assessed by the BSS score was not consistently associated with subjective or objective results. Hip joint degeneration was the most important risk factor for conversion to total hip replacement. In summary, JH as assessed by BSS scores influenced most/all,^68^ some,^42^, ^66^ or none^46^, ^69^ of the hip joint–specific or arthroscopy JH study outcomes.

### Knee Joint–Specific or Arthroscopy JH Study Results

Given the high number of studies, the publication source, purpose, results, and conclusions of knee joint–specific studies are displayed in Table S2. In summary, the JH characteristic as identified by the BSS score was useful to evaluate ACL injury risk,^37^, ^83^ ACL graft injury risk,^9^, ^38^, ^45^, ^48^, ^51^, ^52^, ^59^, ^62^, ^65^, ^71^, ^86^, ^87^ knee function and pain,^55^ plantar flexor neuromuscular activation,^57^ RTS rates,^64^ reduced PROM validity,^67^ knee valgus during drop landings,^75^ rotatory knee laxity,^80^ and anterior tibial subluxation.^89^ In summary, JH as determined by BSS scores influenced most/all,^9^, ^37^, ^38^, ^45^, ^48^, ^51^, ^55^, ^59^, ^62^, ^64^, ^71^, ^75^, ^80^, ^83^, ^88^, ^90^ some,^52^, ^57^, ^67^, ^81^ or none^35^, ^65^, ^72^, ^87^ of knee joint–specific or arthroscopy JH study outcomes.

#### Ankle Joint–Specific or Arthroscopy JH Study Results

The following is a synopsis of ankle joint–specific or arthroscopy JH studies. Baillie et al.^36^ compared clinical assessment findings between elite ballet dancers with and without posterior ankle impingement syndrome (PAIS). A BSS score ≥5 indicated JH; however, as defined, only 1 study subject had JH. The PAIS group completed fewer single leg heel raise repetitions (*P* = .02) and were more symptomatic for perceived ankle instability according to Cumberland Ankle Instability Tool scores (*P* = .004). They concluded that single leg heel raise endurance capacity was lower and perceived ankle instability was greater in participants with PAIS. In a balance training study of patients with chronic ankle instability who either had JH (BSS score ≥4) or did not have JH (BSS score <4), Hou et al.^53^ reported that groups were comparable at baseline except for the JH group displaying reduced posteromedial (83.6 ± 10.1% vs 92.8 ± 12.3%) and posterolateral (84.7 ± 11.7% vs 95.7 ± 8.7%) movement symmetry during Star Excursion Balance Testing (SEBT). After balance training, the JH group had a lower repeat ankle sprain ratio both immediately after training (11.1% vs 23.5%) and at 3 months (16.7% vs 29.4%) after training than the non‐JH group, as well as greater plantar flexor and dorsiflexor strength at both time periods. The JH group also had greater Foot and Ankle Ability Measure (FAAM) sports subscale score improvements. They concluded that because patients with JH and with chronic ankle instability displayed equal or better postural stability and muscle strength improvements after balance training compared with non‐JH patients, balance training prior to surgery might prove particularly effective for them.

Porter et al.^73^ evaluated the clinical outcomes of patients who underwent ankle ligament augmentation reconstruction (LARS) compared with control group subjects based on increased bodyweight or JH (BSS score ≥ 5). From pre‐surgery to 2 years and 5 years post‐surgery, Tegner Activity Scale scores improved in both groups (*P* < .001). Compared with control group subjects, however, Tegner Activity Scale scores were lower in the >90‐kg bodyweight group at both FU periods (*P* < .001), while the JH group had similar scores as control group subjects at both FU periods. Both the >90‐kg bodyweight and JH groups failed to show significant Foot and Ankle Orthopedic Society (FAOS) subscale score improvements compared with control group subjects at both FU periods. There were no recurrences, repeat surgeries, or major complications. They concluded that LARS use was a viable surgical option in patients for whom the Mini‐Brostrom‐Gould (MBG) procedure might be contraindicated. Wang et al.^85^ compared the clinical outcomes and RTS rates between anatomic ankle reconstruction using free tendons and MBG approach use in patients with JH (BSS score ≥4). The FAOS and Karlsson scores improved after surgery in both groups (*P* < .001), with the reconstruction group displaying higher postoperative FAOS‐Sports scores (87.9 ± 8.9 vs 80.5 ± 11.6; *P* = .015) and Karlsson scores (86.9 ± 6.1 vs 82 ± 8.4; *P* = .025) than the MBG group. The RTS rate for preinjury high‐demand sports was higher in the reconstruction group than in the MBG group (73.3% vs 38.9%, *P* = .034). The MBG group also had a higher sprain recurrence rate (22.4% vs 0%, *P* = .027). More patients reported ankle dorsiflexion restriction in the reconstruction group (n = 4; 21.1%) than in the MBG group (n = 1; 2%) (*P* = .019); however, there was no noticeable effect on daily life or sports. They concluded that better clinical outcomes, less sprain recurrence, and a higher RTS rate for preinjury high‐demand sports occurred with anatomic ankle reconstruction using free tendons compared with the MBG procedure in patients with JH. In summary, JH as assessed by BSS scores influenced most/all^53^, ^86^ or none^36^, ^73^ of the ankle joint–specific or arthroscopy JH study outcomes.

## DISCUSSION

The most important study finding was that compared with joint‐specific or arthroscopy JH studies, generalized JH studies, although being published a mean 6 years earlier, more frequently used prospective study designs, had higher evidence levels, and more routinely reported more detailed separate subject age and sex information. Although JH as identified by BSS scores generally influenced the measured outcomes in both study types, greater use of prospective study designs, with higher evidence levels, with more detailed, separate subject age and sex information in joint‐specific or arthroscopy JH studies may strengthen surgical and clinical decision‐making and patient outcome prediction validity.

Overall, generalized JH and joint‐specific or arthroscopy JH studies displayed moderate‐to‐good study quality with low bias risk. Because female and male as well as younger and older patients often possess differing responses to surgery and rehabilitation, for surgical and clinical decision‐making purposes, it is vital to obtain population‐specific sex, age, activity level, expectation, and behavioral information. For arthroscopy patient outcome studies that use BSS scores to contribute more strongly to surgical and clinical decision‐making, it is important that prospective study designs with more detailed subject information be used.

Most studies established a JH threshold using BSS scores of ≥4 or ≥5; however, 3 knee‐specific studies used BSS scores and the coexistence of ≥10° genu recurvatum at 1 knee,^37^, ^48^, ^65^, ^67^ or an added injury point allowance^44^ as knee JH indicators. For ACL injuries, Zsidai et al.^9^ recommended thorough clinical examination, diagnostic imaging, and documentation of both the aggregate BSS score for overall JH assessment and knee hyperextension magnitude. To better address the adolescent athlete target population, several knee arthroscopy studies further established high reinjury risk as participation in pivoting or cutting sports and the presence of a ≥2 clinical pivot‐shift grade.^37^, ^48^, ^65^, ^67^ In combination with a BSS score ≥4, ≥10° genu recurvatum, a positive clinical pivot shift test, and an excessive posterior tibial slope may better identify individuals at greater ACL graft injury risk.^37^, ^48^ Some have suggested that these individuals might benefit more from combined intra‐articular ACL reconstruction, extra‐articular anterolateral augmentation,^37^, ^48^ and a slower rehabilitation progression.^91^ In this context, the target population was predicted from a combination of age, behavioral expectation, aggregate BSS score threshold, a specific JH and joint laxity predisposition, and specific osseous morphology.^37^, ^48^, ^65^, ^67^ Because the BSS score was developed as a large‐group, generalized JH epidemiological screening tool, it is not sufficient for confirming joint‐specific JH. In addition to age, gender, relevant joint‐specific morphology, and patient behaviors, a combination of BSS scores and valid, reliable joint‐specific JH and capsuloligamentous joint laxity tests can better identify which patients might benefit most from arthroscopic surgery and a particular rehabilitation plan. Given the distinct age‐related developmental and behavioral differences that occur at different life periods, particularly during adolescence,^92^ for surgical and clinical decision‐making purposes, patient demographic information should be as individualized and detailed as possible.

Post–arthroscopic surgery patient outcome success generally includes rehabilitation efficacy.^93^, ^94^, ^95^ How composite BSS scores for generalized JH relate to joint‐specific or arthroscopy JH clinical examination findings and radiographic osseous morphology information is important. However, for surgical and clinical decision‐making purposes in the “high injury or reinjury risk” adolescent athlete population,^48^ behavioral factors such as athletic self‐identity,^96^ fear,^97^ confidence,^98^ cognitive appraisals,^99^ and health locus of control^100^ may also be important to know.

To foster cost effective osteoarthritis prevention and wellness programs for both high joint injury (primary prevention) and reinjury risk (secondary prevention) patient populations,^101^, ^102^, ^103^ improved interdisciplinary collaboration is needed.^104^, ^105^ Many of the perceived ACL graft reinjury “risk” factors reported by knee‐specific studies^37^, ^48^, ^65^, ^67^ may be of even greater value for primary injury prevention. Would adolescent athletes with a severe posterior tibial slope, a BSS score ≥5°, and ≥10° genu recurvatum with a noncontact ACL rupture family history who participate in pivoting sports not also possess greater primary ACL injury risk? Much of the same information used to predict high ACL graft reinjury risk might be equally, if not more, valuable in preventing primary, noncontact ACL injury.^106^

Perhaps the breadth of adolescent patient data obtained from different health care providers should be better aggregated, disseminated, and applied through more focused primary (native ACL) and secondary (ACL graft) injury prevention programming. Although already collected, much of this information too often resides in isolated “data silos” not available to many of the interdisciplinary health care team members who could collaboratively develop injury prevention programs.^107^ Similar to the data obtained from interdisciplinary preseason physical examinations, for injury prevention program development patient data obtained from individual health care provider encounters might be better aggregated to improve health behaviors earlier in high‐injury‐risk populations, better facilitating morbidity‐free survival later in life^102^ and reducing chronic disease and disability prevalence over a lifetime.^102^, ^104^

Public health programs are assessed based on whether the improvements they provide justify their cost.^103^ This process involves the following: (1) a low cost; (2) high downstream health care expenses such as emergency room visits or orthopaedic surgery and rehabilitation; and (3) the ratio of how many people need to receive the prevention treatment to reduce costs.^105^ With high target population identification accuracy, the number needed to treat should be sufficiently low to enhance program efficacy.^101^, ^103^

For example, low‐cost therapeutic exercise programs that reduce senior patient falling rates and decrease downstream health care costs represent one of the best public health programs for return on investment.^101^ Shifting investments from the secondary care for patient injuries sustained from a fall to a stronger primary prevention emphasis generates substantial investment return, which is further increased when the most at‐risk population has been accurately identified and treated.^101^

Getting back to primary ACL injury prevention, many female adolescent athletes specialize in a single sport year‐round under coaches with limited or no injury‐prevention knowledge.^108^ Over the past 2 decades, United Kingdom knee surgeons have experienced a 29x increase in the number of ACL surgeries that they have performed.^108^ In addition to previously mentioned clinical, morphological, behavioral, and patient expectation factors, a combination of patient and family medical histories, high training volume, high competition level, and poor compliance with injury prevention interventions may further increase primary or secondary injury rates and should also be factored into primary ACL injury prevention models.^9^, ^108^, ^109^

Several studies provided a useful knee joint–specific template for validating arthroscopy and rehabilitation intervention efficacy in adolescent athletes.^37^, ^48^, ^65^, ^67^ Criteria developed for different joints and different target populations may differ based on subject age, sex, unique joint‐specific morphology, tissue contributions to biomechanical joint loading, target population activities, behaviors, and expectations. Although BSS scores in combination with joint‐specific clinical examinations can provide helpful information, to more accurately predict patient outcomes and future injury risk, additional factors may need to be considered.

### Limitations

This study is not without limitations. Only English‐language studies from 3 databases contributed to this review. Articles published in other languages or databases may have been missed. Additionally, because this study was designed to capture the breadth of BSS score use to evaluate the influence of JH, some JH study areas may have lacked content‐specific depth. Lastly, accepted studies were determined solely by a group of 3 reviewers. Therefore, some reviewer bias may have influenced which studies contributed to this review.

## CONCLUSIONS

Generalized JH studies had more prospective research designs, had higher evidence levels, and more frequently reported separate subject age and sex details. Greater use of these characteristics in joint‐specific or arthroscopy JH studies may strengthen surgical and clinical decision‐making and patient outcome prediction validity.

## SUPPORTING INFORMATION

Additional supporting information can be found online in the Supporting Information section.

## DISCLOSURES

The authors (J.N., E.G., J.L., J.G., R.K., J.R.) declare that they have no known competing financial interests or personal relationships that could have appeared to influence the work reported in this paper.

## Supporting information

Supplementary Material
