# Supplementary material for: Beighton Scoring System Use in Generalized Joint Hypermobility Studies Has Greater Scientific Rigor Than Joint‐Specific or Arthroscopy Joint Hypermobility Studies
Source: Arthrosc Sports Med Rehabil. 2026 May 4;8(2):e70000. doi: 10.1002/ars2.70000 (PMC13307201; doi:10.1002/ars2.70000)
Supplement: Supplementary file 1 — Supplementary Material [file ARS2-8-e70000-s001.zip › APPENDIX_BSS_ZZZ7_ASMAR_Z7.pdf]

APPENDIX I: ACL-QoL = Anterior Cruciate Ligament Quality of Life Score; ACL-RSI = Anterior Cruciate Ligament-Return to Sport after Injury Score; ADL = activities of daily living; ALL = anterolateral ligament; AP = antero-posterior; AROM = active range of motion; ATT = anterior tibial translation; ATTp = anterior tibial translation peak; ATTd = anterior tibial translation distance; ATS = anterior tibial subluxation; BC = Brighton Criteria; BESS = balance error scoring system; BMI = body mass index; BPTB = bone-patellar tendon-bone; BSS = Beighton Scoring Scale; BW = bodyweight; CAI = chronic ankle instability; CAIT = Cumberland Ankle Instability Tool (CAIT); CCI = co-contraction index; CI = confidence interval; COPL = center of pressure path length; CT = computerized tomography; DLVJ = double-leg vertical jump; ER = external rotation; FAAM = Foot and ankle ability measure; FAI = femoro-acetabular impingement; FAOS = foot and ankle outcome score; FPI = foot posture index-6; FU = follow-up; GHJ = glenohumeral joint; GR = genu recurvatum; JH = hypermobile or hypermobility; HA = hip arthroscopy; HTA + LET = hamstring autograft + lateral extra-articular tenodesis; HOS-ADL = Hip Outcome Score-Activities of Daily Living; HOS-SSS = Hip Outcome Score-Sport-Specific Subscale; HR = hazard ratio; HRQoL = health-related quality of life; HSS Pedi-FABS = Hospital for Special Surgery Pediatric Functional Activity Brief Scale; HTA = hamstring tendon autograft; IKDC = International Knee Documentation Committee; IPT = iliopsoas tendinitis; KOOS = Knee Injury and Osteoarthritis Outcome Score; iHOT-12 = International Hip Outcome Tool - 12; LARS = ligament augmentation reconstruction system; LCL = lateral collateral ligament; LE = lower extremity; LKS = Lysholm Knee Score; LLAS = lower limb assessment score; LLS = ligament or ligamentous laxity scale; LSI = limb symmetry index; MBG = modified Broström Gould; mHHS = Modified Harris Hip Score; MCID = minimally clinical important difference; MS = musculoskeletal; MTPJ = metatarsophalangeal joint; MVE = maximal volitional exertion; NAHS = Non-Arthritic Hip Score; NCAA = National Collegiate Athletic Association; NJH = non- joint hypermobile or non-joint hypermobility; NPV = negative predictive value; PAIS = posterior ankle impingement syndrome; Pedi-IKDC = Pediatric International Knee Documentation Committee (Pedi-IKDC) Subjective Knee Evaluation; PPV = positive predictive value; PROM = patient reported outcome measurement; PTS = posterior tibial slope; QA = quadriceps autograft; ROM = range of motion; RR = relative risk; RTP = return to performance; RTS = return to sports; SANE = single assessment numeric evaluation; SEBT = star excursion balance test; sEMG = surface electromyography; SLDL = single-leg drop landing; SLHR = single leg heel raise; SLS = single leg squat; TASS = Tegner Activity Scale Score; THR = total hip replacement; UCLA = University of California Los Angeles; shoulder score UK = United Kingdom; VAS = visual analog scale; VB = volleyball; %MVE = percentage maximal volitional effort;

**Generalized JH**						
**Study**	**Purpose**	**Subjects/Methods**	**Tools**	**BSS Criteria**	**Results**	**Conclusion**
Armstrong R & Greig M[34]	Determine efficacy of BSS score use as a predictor of BC components, considering the influence of gender and sports participation.	65 female and 38 male rugby players; 61 netball players; 42 female dancers; 40 female and 40 male controls.<br>:	BSS score and the BC were used to assess joint H.	≥ 4/9	BSS were predictive of arthralgia (p = .002), dislocation and subluxation (p = .048). In the pooled analysis they were predictive of dislocation and subluxation (p = .047) in males and arthralgia (p = .001) in females. BSS score was a predictor of arthralgia in rugby (p = .003) and in controls (p = .012).	The potential of the BSS to predict joint arthralgia and dislocation/ subluxation may allow clinicians to implement effective injury prevention strategies.
Bronner S & Bauer NG.[39]	Examined injury risk factors in pre-professional modern dancers.	Screening and injury data of 180 university modern dance program students were reviewed over 4-yrs of training. Four categorical risk factors were studied: i) jH; ii) dance technique motor-control; iii) muscle tightness; iv) previous injury.	Dancers were divided into 3-groups based on predictor scores. Exposure was based on hours of technique classes/week. Negative binomial log-linear analyses were conducted with the four predictors.	5-9/9 = high; 3 or 4/9 = medium; 0-2/9 = low	Dancers with low and high BSS scores were 1.43X and 1.22X, respectively more likely to sustain injury than dancers with mid-range scores (p ≤ .03). Dancers with better technique (low or medium scores) were 0.86X and 0.63X less likely to sustain injury (p = .013 and p < .001) compared to those with poor technique. Dancers with one, or 2-4 tight muscles were 2.7X and 4.0X more likely to sustain injury (p ≤ .046). Dancers who sustained 2-4 injuries over the previous year were 1.38X more likely to sustain subsequent injury (p < .001).	Dancers with these risk factors may benefit from injury prevention programs.
Bukva B, et al.[40]	Evaluated relationship between JH in artistic gymnasts and injury rate over a 1-year season.	24 (11-26 years old), members of Qatar National Team in artistic gymnastics	BSS score and seasonal injury survey. Gymnast traits including age, sex, training/day, training years, and their relation to injury rate were evaluated.	≥ 5/9	Low back pain was most common, followed by knee, shoulder, hip and ankle injuries. There was a strong correlation between training years and injury rate (p < .001). There was no relationship between training hours/week, H and injury number (p > .05).	There was no relationship between JH and injury rate. Total training time contributed more strongly to injury rate.
Clinch J, et al.[43]	Describe the prevalence and pattern of JH in 14-year-old UK children.	6,022 children, mean age 13.8 years were evaluated for JH	Objective physical activity measures were measured with accelerometry. Puberty and	≥ 4/9	JH (BSS ≥ 4) in girls and boys was 27.5% and 10.6%, respectively. 45% of girls and 29% of boys had finger JH. There was a positive relationship between H in girls	The prevalence of JH in UK children was high suggesting that a BSS cutoff ≥ 4 was too low or not appropriate

			socioeconomic status data were collected. Prevalence rates were calculated. Variable associations with H were assessed.		and physical activity, BMI, and maternal education. No associations were seen in boys.	for subjects with developing MS systems. Results provided a platform to evaluate JH criteria and key clinical features (including pain) in the pediatric population.
Collinge R & Simmonds JV[2]	Evaluate if JH was an injury risk factor in a professional football team.	33 males 18-35 years were assessed using a H screen and a seasonal injury audit.	Primary objectives were to estimate H prevalence and to audit injuries sustained over a season. Secondary objectives were to relate the injury audit findings and JH levels to time missed from injury, assessed by training days and competitive first team games missed after MS injury.	≥ 4/9 = JH; ≥ 7/9 = excessive JH	JH was between 21-42% depending on JH cut-off score. Similar injury rates were found in the JH and non-JH participants (6.2 vs. 6.3 injuries/1000 hours exposure respectively). Once injured, the JH group missed more games (12/season vs. 5/season for non-JH) and training days (71 days/season vs. 31 days/season for NH).	JH prevalence was dependent upon which cut-off score was used. RTS in athletes with may be extended to minimize the potential risk of re-injury and limit the socio-economic costs associated with time out of competition.
Decoster LC, et al.[44]	Evaluated JH prevalence in adolescent athletes during pre-participation physical exams.	264 athletes (150 male, 114 female; mean age = 15.5 years).	JH scores were determined	≥ 5/9 (included an additional injury allowance point if one side of a bilateral test had sustained an injury	32 subjects were JH, with another 2 screening JH + by injury allowance, for a total of 34 JH athletes (12.9%). There was a significant difference between sexes (p < .001), with 25 female (22%) and 9 male subjects (6%) testing +.	JH prevalence and the significant sex difference found in this group of adolescent athletes were similar to non-athlete populations of comparable age. Prospective studies are warranted to investigate this question before we can justify depriving H youths of the benefits of regular or strenuous exercise.
Frisch A, et al.[47]	Used pre-season examinations to identify player-related risk factors for injuries in youth soccer.	Male under 15, 17, and 19 year of age players from a regional soccer school (n = 67).	Physical fatigue, emotional stress and injury history (survey), anthropometric variables, JH, LE coordination (hop tests), aerobic fitness (shuttle run test), knee extensor and flexor muscle strength (isokinetic tests), balance (force plate tests), and explosive strength (jump tests on force plate). Exposure and soccer-related injuries (n = 163) were recorded during the season (44 weeks).	≥ 4/9	Total injury incidence was 10.4 injuries/1000 hours and was higher in games than in training [RR = 3.3; CI 95% (2.39; 4.54. p < .001). LE injuries were most frequent (87%). Acute contact injuries represented 37%, while intrinsic (noncontact and chronic) injuries amounted to 63%. Only physical fatigue was associated with injury.	A single preseason test session may be of limited value in the framework of an injury prevention strategy.
Handziikova et al.[49]	Attempted to determine if JH influenced ACL injuries.	Evaluated biomechanical differences between 15 subjects with JH and 27 subjects without H. Participants were regular cutting sport participants.	3D ankle, knee, hip, pelvis, and trunk angles were determined during the first 100 ms after unanticipated side-step cutting initial contact. Data from	≥5/9 (women), ≥ 4/9 (men)	JH group had lower minimum knee valgus angles with a mean difference of 3.5° (p = .03, Hedge g = .69) and greater peak knee ER with a −4.5° mean difference (p = .04, Hedge g = .70) during dominant leg cutting, and lower peak ankle	Despite moderate cutting kinematic group differences, isolated kinematic variables were considered to serve crucial roles in non-contact knee and ACL

			the JH and non-JH groups were compared with sex as a confounder.		plantar flexion angles with a 4.5° mean difference (p = .03, Hedge g = .73) during non-dominant leg cutting compared with NH group.	injury mechanisms
Hawke F, et al.[50]	Explored relationships between foot posture, flexibility, and body mass in children.	30 healthy, asymptomatic children (20 girls, 10 boys) aged 7- 15 years, mean age (SD) of 10.7 (2.3) years	Height, weight, BMI, FPI, BSS, LLAS score and ankle lunge angle. Correlation analysis was used to explore variable relationships.	≥ 5/9	Higher FPI was associated with higher H (r = .44, p = .01); greater lunge angle was associated with higher H (r = .40, p = .02) and LLAS (r = .42, p = .02) scores; older age was associated with higher BMI (r = .52, p = < .01) and with lower H (r = −.41, p = .024) and LLAS (r = −.40, p = .03) scores; and higher H was associated with higher LLAS (r = .85, p = <.01).	Children with a more pronated foot type had greater LE whole-body flexibility, but not greater ankle flexibility. There was strong agreement between LE and whole-body flexibility.
Johnson AP, et al.[54]	Attempted to validate LLAS against JH score within an adult elite soccer population.	36 male, professional soccer athletes between 18-37 years of age.	Sensitivity, specificity, PPV, NPV and Spearman's rank correlation between the LLAS and JH.	≥ 5/9	Strong correlation between LLAS and JH (ρ = .732; p < .001). The LLAS had sensitivity of 67%. Specificity of 94% occurred when a ≥ 4 cut-off was used. This cut off point yielded moderate PPV (50%) and excellent NPV validity (97%).	LLAS was a valid test for identifying LE JH within an adult male soccer population when a ≥ 4/12 cut-off was used.
Juul-Kristensen B, et al.[56]	Evaluated knee muscle activity and static postural sway in girls with JH.	16 girls with JH and 11 girls with non-JH aged 14 years were randomly recruited.	Static two-legged balance test with eyes open and eyes closed and a one-legged stance test with eyes open. Postural sway (COPL) was sEMG from the quadriceps, hamstrings, and gastrocnemius muscles was expressed as a %MVE and the CCI of muscle activity was calculated. Knee function was self-reported using the KOOS-Child survey.	≥ 6/9 and one JH knee	Girls with JH had a lower lateral HQ CCI and a higher medial/ lateral HQ CCI ratio in all balance tasks. Group mean sEMG varied from 1.3 %MVE in Q (both eyes open) to 15.7 %MVE in gastrocnemius (1 eye open). Girls with H had larger postural sway length than girls with non-JH (both eyes closed)(COPL 1.64 vs. 1.37 m/min, p < .001).	Girls with JH and at least one knee with GR performed static balance tasks with higher medial knee muscle activity relative to the lateral activity, and larger postural sway when vision was eliminated.
Konopinski M, et al.[60]	Compared injury incidence between elite soccer players with and without JH.	80 players from 3 English championship soccer teams were followed over 1-season.	Player exposure and time-loss injuries were recorded.	≥ 4/9	Injury incidence was 9.2 ± 10.8 injuries/1000 hours. JH prevalence was 8.8%. Players with H had higher injury incidence (mean [95% CI difference, 5.2 [0.9-2.7] injuries/1000 hours; p = 0.06). Overall training exposure related to injury risk (p < .001). Players with no JH had a lower injury risk (p = 0.11), suggesting that H might predispose injury risk.	Players with JH displayed a trend towards increased injury risk. Training exposure was a significant injury risk factor.
Krivickas LS & Feinberg, JH[61]	Evaluated muscle tightness, and ligamentous laxity to determine if they were associated with LE injury incidence.	201 college athletes	BSS, LE injuries incurred during practice and play were recorded during the following year.	0-3/9 = NH; 4-6/9= JH; 7-9/9 = extreme JH	71 athletes sustained 115 injuries. For each point on the 9-point LLS (9 = hyperlax), injury risk decreased 16%. For each point on the 10-point muscle-tightness scale (10 = all muscles tight), injury risk increased 23%. The two scores were moderately inversely correlated (Spearman's rho = -.3; p < .001). Women had greater laxity than men (3.3 +/- 2.2 vs 1.8 +/- 2.0; p < .001) and lower overall muscle tightness (1.5 +/- 1.6 vs. 3.5 +/- 2.1; p < .001). Among women LE injury rate was unrelated to LLS scores	Tight ligaments and muscles were related to injury in men, but not women. A preseason flexibility program may decrease injuries in male college athletes.

					or to flexibility. Among men, LE injuries were associated with lower LLS scores (p = .008) and with greater muscle tightness (p = .04).	
Nicolay RW, et al.[70]	Studied JH as a predisposing risk factor for injury in NCAA Division I football players.	JH scores were collected for 73 athletes during pre-season physical exams.	Athlete age, height, weight, and playing position, were recorded. Players were followed for 2 years, and the # of MS issues, injuries, treatment episodes, days unavailable, and surgical procedures were recorded.	≥ 4/9	Overall mean H was 1.4 ± 1.5; 7 players (9.6%) had JH. During the study period there were 289 injuries. The mean # of treatments/ athlete was 77 ± 71 (range, 0-340), and the mean # of days unavailable was 67 ± 92 days (range, 0-432 days). There were 23 athletes who required 25 operations, the most common procedure was arthroscopic shoulder stabilization (n = 6). The # of injuries/athlete was not different between the JH and NJH groups, nor were there group differences for the # of treatments received, or surgery rates.	A preseason JH diagnosis did not place NCAA football players at a greater injury risk.
Rejeb A, et al.[74]	Examined the association between JH and injury rates in Middle Eastern male youth athletes.	226 athletes (mean age: 14.2 years; range: 10-18) involved in 15 different sports were grouped into contact and non-contact sports.	Subjects underwent a JH screen and a seasonal injury audit, athletes' anthropometric characteristic (weight, height and BMI) were recorded.	0-9/9	226 athletes sustained 596 injuries and 75% reported ≥ 1 injury over a seasonal injury audit. Contact sport players were injured more often than players in non-contact sports (more frequent injuries than injury-free time in contact sports; 127 days (95% CI 93-160) vs 176 days in non-contact sports (95% CI 118-234) (p < .001). Survival analysis showed that JH was not associated with injury HR = 1.004 (95% CI 0.95-1.06) in the overall cohort. JH score was associated with a greater injury risk in contact sports (HR: 1.29; 95% CI 1.05-1.59; p=.015).	Greater JH and contact sport involvement influenced injury risk. As an injury pre-emptive measure preseason JH documentation should be considered for contact sport athletes
Schmidt H, et al.[76]	Assessed JH prevalence in elite adolescent athletes, and the association of JH with pain, function, HRQoL, and MS injuries.	132 athletes (36 boys, 96 girls; 14.0 ± 0.9 years of age), including ballet dancers (n = 22), TeamGym gymnasts (n = 57), and team handball players (n = 53).	MS injuries, and HRQoL were assessed with PROM and part of physical performance was assessed using postural-sway and single-legged hop-for-distance tests.	≥ 4/9, ≥ 5/9, ≥ 6/9.	Overall prevalence rates for JH scores of 4/9, 5/9, and 6/9 were 27.3%, 15.9%, and 6.8%, respectively, with a higher prevalence of 4/9 in ballet dancers (68.2%) and gymnasts (24.6%) than team handball players (13.2%). There was no difference in LE function, injury prevalence and related factors (exacerbation, recurrence, and absence from training), HRQoL, or hop test distances for JH or NH. However, the JH group had larger COPL during sway tests.	Ballet dancers had greater JH than team handball players and compared to the general adolescent population. The JH group had larger sway in the balance tests but this did not have an association with injuries or HRQoL. Ankle injury risk due to larger sway for the JH group warranted further study.
Skwiot M, et al.[77]	Assessed JH prevalence in jazz dancers and their relationship to other potential injury risk factors.	77 dancers from the Polish Dance Theater were examined (58 female and 19 male), between 18-25 years of age.	Structured interview, JH, modified Grahame & Hakim questionnaire.	≥ 4/9	JH prevalence differed depending on which criteria were adopted (p = 0.001), with BSS, modified Grahame & Hakim questionnaire, and Sachse's criteria identifying 64.9%, 74% and 59.7% of the sample as JH respectively. JH was more prevalent in women than men.	There was a significant prevalence of JH in jazz dancers. There is a need for more unified diagnostic criteria.
Smth R, et al.[78]	Evaluated the incidence of JH in young female netball players to determine the relation between	200 < 16 year old players from a local suburban netball association	Player profiles and sport injury details related to netball and other sports, and protective	0-2/9 not JH; 3-4/9 moderate JH; 5-9/9 distinct JH	21% of subjects with JH = 0-2 had sustained previous netball injuries compared with 37% with JH = 3-4, and 43% with JH = 5-9. These differences were significant	H was associated with increased injury prevalence. Targeted intervention may reduce injuries.

	JH, previous injuries sustained in netball or other sports, and protective equipment use.		equipment use were gathered using a subject reported survey.		(p < .025). Ankle (42%), knee (27%), and finger (15%) injuries were most common. 39 players (19%) wore protective equipment, and within this group 30 (77%) had sustained previous injuries. No association was detected between JH and non-netball related sport injuries.	
Soper K, et al.[26]	Evaluated the influence of JH on functional movement control and explored whether dysautonomia symptoms existed.	27 elite netballers (14-26 years)	Functional movement control was measured using force platform posturography and the Star Excursion Balance Test (SEBT).	≥ 4/9 = JH; 4-6/9 = high JH; 7-9/9 = distinctly JH	JH prevalence was 63% (n = 17) (BSS ≥ 4/9). Symptoms of dysautonomia were minimal. Participants with JH had less postural instability on the functional tests.	High JH prevalence was observed with a trend towards impaired functional movement control.
Sueyoshi T, et al.[79]	Investigated JH and incidence of ligament injuries in high school female volleyball players.	47 subjects were tested for JH	Categorized injury and non-injury groups based on past ligament injury history. Further divided based on whether they had a single injury or multiple injuries.	0-9/9	The injury group had higher mean JH scores than the non-injury group (2.4 ± 1.42 vs. 1.24 ± 1.09, p = .006). 11 subjects in the injury group had multiple or recurrent injuries and scored higher than the remaining 19 injury group subjects who had only sustained a single injury. Mean JH scores were 3.18 ± 1.47 vs. 1.95 ± 1.22, p = 0.02.	JH female athletes may be more prone to ligament injury and potentially to recurrent ligament injuries.
Tobias JH, et al.[82]	Studied whether JH in childhood is a risk factor for subsequent MS pain.	2901 (1267 boys, 1634 girls) mean 17.8 years of age	JH was determined according at mean 13.8 years of age; MS pain was determined using a survey at mean 17.8 years.	≥ 6/9	4.6% had JH at mean 13.8 years. MS pain was most common at the low back (16.1%), shoulder (9.5%), upper back (8.9%), knee (8.8%), neck (8.6%), and ankle/foot (6.8%). JH was associated with increased pain at the shoulder (OR = 1.68, 95% CI = 1.04; 2.72), knee (OR = 1.83, 95% CI = 1.10, 3.02; and ankle/foot (OR = 1.82, 95% CI = 1.05, 3.16. Associations between JH and knee pain showed higher OR in obese participants (OR = 11.01) compared with non-obese participants (OR = 1.57) (p = .037).	JH was a MS pain risk factor at the shoulder, knee, and ankle/foot. Relationships were strongest with obesity, consistent with a causal pathway whereby JH leads to pain at sites exposed to the greatest mechanical forces.
Van Meulenbroek et al.[84]	Studied whether Dutch adolescents with asymptomatic JH had lower physical activity, muscle strength, motor performance, and perceived harmfulness compared to NJH adolescents.	17.0 ± 3.8 years of age subjects with JH or NJH.	VAS pain level, perceived harmfulness, isokinetic knee strength/endurance, single leg hop for distance, movement accelerometry.	≥6 (< 18 years of age); ≥5 (≥ 18 years of age)	Adolescents with JH had increased knee extensor torque/BW compared to NJH subjects when controlled for age and BW.	Adolescents with JH had greater peak knee extensor torque/BW than NJH adolescents.
van Rijn R & Stubbe J[85]	Studied whether JH as defined by different threshold cut-off values was a potential risk factor for injuries in first-year pre-professional dancers.	Four cohorts (N = 185), mean age 19.1 ± 1.3 years, were screened for MS function at the start of the academic year	All completed monthly physical and mental health surveys using the performing artist and athlete health monitor (PAHM).	0-9/9	Overall mean JH was 2.8. The 1-year injury incidence proportion was 67.6% (n = 125), 43.2% (n = 80), and 54.6% (n = 101) for all complaint injuries, substantial injuries, and time-loss injuries, respectively. There was an association between previous long lasting injury in the past year and the 3 injury definitions (p < 0.05).	Dancers were at high risk for injuries and H. However, these variables were not associated with each other. Health professionals should take injury history into account when assessing dance students, because this variable is associated with increased injury risk.
Zhong G, et al.[89]	Evaluated if the active 3D knee kinematics of	489 subjects divided into JH (n = 54) and NJH	Survey participant demographic	≥ 4/9	JH prevalence was 11%. Subjects with H had greater AP AROM (p =	Poor AP AROM stability may play

	college students with JH was poor compared to that of NJH people.	(n = 435) groups.	characteristics and MS disorder symptoms. 3D gait analysis system was used to collect knee kinematic parameters during treadmill walking.		.026). Subjects with JH had greater knee flexion at terminal stance (p = .039) and greater ATT during most of the gait cycle (p < .05). A greater external angle was observed in JH group during mid-stance (p = .008).	contribute to knee joint instability, potentially resulting in subsequent ACL deficiency and knee OA development in people with JH.
**Shoulder Joint-Specific or Arthroscopy JH**						
**Study**	**Purpose**	**Subjects/Methods**	**Tools**	**Criteria**	**Results**	**Conclusion**
Cameron KL, et al.[41]	Examined the relationship of sex, JH, and a history of JH instability within a young, physically active cohort and to describe JH incidence within this population.	1050/1311 (80%) of an entering college freshman class.	JH, history of JH instability was identified via a baseline survey.	≥ 2 (95th percentile cut-off criterion)	Most (78%) had no JH signs. 11 (1.5%) had H ≥ 4. Logistic regression revealed a relationship between JH and JH instability history (p = .023). When gender and race were controlled, those with a total JH ≥ 2 were 2.5X as likely (OR = 2.48, 95% CI = 1.19, 5.20, p = .016) to have JH instability history. A relationship was observed between gender and most individual BSS score items. Although women had higher total JH scores than men, gender (p = .658) and race (p = .410) were not related to a history of JH instability when other variables were controlled	JH and a history of JH joint instability were associated.
Khan U, et al.[58]	Studied the causes and management of recurrent GHJ instability post-Latarjet surgery were evaluated and revision surgery outcomes.	16 patients (12 male and 4 female) who underwent revision surgery for recurrent GHJ instability post-Latarjet surgery. 11 were athletes (9 professional, 2 amateur). Mean age age at revision was 29.9 ± 8.9 years.	Data were collected over a 5-year period including demographics, clinical exam, recurrent instability cause, revision surgery indications, intraoperative analysis, revision surgery outcomes and RTS.	0-9/9	Revision indications were anterior instability (n = 11), posterior instability (n = 4), and both anterior and posterior instability (n = 1). Of the anterior instability cases, 54.5% were due to coracoid non-union and 36.4% were due to capsular failure (re-tear). All posterior instability cases had posterior capsulolabral injuries, and the mean JH score in this group was ≥ 6. One patient had a failed Latarjet procedure with coracoid non-union and a posterior labral tear.	Coracoid non-union was the most common cause of GHJ instability recurrence. Patients who returned with posterior instability had a high JH prevalence.
Lim JR, et al.[63]	Studied bipolar bone defect characteristics in the presence of excessive JH and clinical outcomes based on the on-track/off-track theory.	81 patients who had undergone arthroscopic Bankart repair, with JH (n = 33) or NJH (n = 48). Additional remplissage was performed for cases with off-track or positive engagement test in borderline on-track lesions.	Bipolar bone lesions were assessed using preoperative 3D CT. Functional outcomes at 2-year FU were assessed using recurrence rate, Subjective Shoulder Value, Rowe score, UCLA shoulder score, AROM, and the sports/ recreation activity level.	≥ 4/9	Group glenoid bone defect frequency difference differences were not evident (H = 14.1%, NJH = 14.4%). Off-track lesions were found in 39.4% (13/33) of the JH group and 14.6% (7/48) of the NJH group (p = .011). Mean Hill-Sachs interval to glenoid track ratio was 83.1% in the JH group and 75.2% in the NJH group (p = .021). Additional remplissage was more frequent in the JH group (48.5%; 16/33) than in the NJH group (16.7%; 8/48) (p = .002). No difference was observed for group shoulder function scores and injury recurrence rates.	Patients with anterior shoulder instability and excessive joint H had wider Hill-Sachs lesions and more off-track lesions despite the lack of a glenoid bone defect frequency difference. Hill-Sachs lesion differences were not related to group functional outcome differences.
**Hip Joint-Specific or Arthroscopy JH**						
**Study**	**Purpose**	**Subjects/Methods**	**Tools**		**Results**	**Conclusion**
Chandrasekaran S, et al.[42]	Reported patterns of clinical presentation, intra-articular derangements, radiological associations in adolescent patients ≤ 18 years of age.	Patients had undergone HA for labral tears that had failed non-operative management with ≥ 2 year FU.	Demographics, examination, radiological and intra-operative findings, intraoperative procedures performed, PROM	≥ 4/9	102 patients satisfied the inclusion criteria, 90 (88.2%, 77 females and 13 males) had ≥ 2-year FU. Females had increased ER in flexion (58.9° vs. 50.0°, p = .041). 68/77 (88.3%) females had a JH score ≥ 4 compared to 6 in males (46.2%) (p < .001). There	HA was associated with improved outcomes, reduced pain, and high satisfaction scores at ≥ 2-year FU. The labral injury pattern was different in males and

			(mHHS, NAHS, HOS-SSS, HOS-ADL, VAS score for pain, and patient satisfaction.		was no distinct pattern within groups or between genders for radiological acetabular coverage markers, depth, or version and femoral cam size. Mean femoral anteversion was 15.7° (females) and 11.3° (males). Females had smaller labral tears (1.73 vs. 2.34 acetabular clock face hours, p = .028). Females more often needed capsular plication and iliopsoas fractional lengthening (88.3% vs 46.2%, and 77.9% vs 38.5%, respectively). PROM improved for both males and females (p < .01), but females had lower pre-operative and post-operative scores. Mean patient satisfaction score was 8.29/10. Five patients (5.56%) required revision.	females dictating the HA approach. Females more often required capsular plication and iliopsoas release to address soft-tissue laxity and impingement.
Firat A, et al.[46]	Reported clinical results of a standardized extended interportal capsulotomy during HA.	Patients 8-60 years of age who had failed non-operative treatment, with at least a 2-year FU.	Data regarding age, sex, operation date, BMI, but also H score, presence of postoperative pudendal nerve damage, and revision for any reason were gathered from patients' records. All patients were evaluated pre-operatively with VAS pain, HOOS, and the mHHS.	0-9/9	90 patients (37.9 +/- 9.8 years of age)(58.9% male) were studied. The most frequent surgical indication was isolated FAS lesion (73.3%), followed by FAI associated with a labral tear (12.2%), an isolated labrum tear (10.0%), synovitis (3.3%), and a loose body (1.1%). Mean FU was 39.3 months. Most patients had uneventful surgeries (76.7%), while there were 3 sciatic nerve neuropraxia cases and 12 pudendal nerve neuropraxia cases. Two patients underwent revision. Comparison between pre- and post-operative clinical scores showed improvement with a final mHHS mean value of 67.7 +/- 18.2, a HOOS value of 74.1 +/- 13.2, and a VAS pain score of 1.3 +/- 1.2.	Few patients had JH ≥2. HA with a standardized and unrepaired, extended interportal capsulotomy was safe with satisfactory mid-term results and high patient satisfaction. At ≥ 2 year FU patients had improved clinical scores and a low revision rate.
Maldonado DR, et al.[66]	Reported PROM at ≥ 2-year FU in patients with JH who underwent HA for symptomatic labral tears and FAI and compared clinical results with a matched-pair NH control group.	Patients who received primary HA for symptomatic labral tears and FAI. Inclusion criteria included pre-operative ≥ 2-year FU scores for PROM: mHHS, NAHS, and VAS for pain.	JH and NH control groups were created. Patients were matched in a 1:2 ratio via propensity score matching according to age, sex, BMI, Tonnis grade, and preoperative lateral center-edge angle.	≥ 4/9	57 patients with JH were matched with 88 controls. Age, gender, BMI, and FU times did not differ (p > .05). Pre-operative radiographic measurements showed no group differences. The JH group received more capsular plications (p = .04). At ≥ 2-year FU PROM improvements were similar between groups for mHHS, NAHS, HOS-SSS, and VAS pain scores (p > .05). Groups also had comparable MCID and PASS rates for mHHS, HOS-SSS, and iHOT-12.	Patients with JH had favorable outcomes after HA for symptomatic FAI and labral tears with appropriate labral and capsular management at ≥ 2-year FU. Compared with a pair-matched control group without H, results were comparable for mHHS, NAHS, HOS-SSS, and VAS and % who reached PASS and/or MCID for mHHS, HOS-SSS, and iHOT-12.
Mojica ES, et al.[68]	Studied whether increased JH was associated with a more frequent iliopsoas tendinitis (IPT) in post-operative HA patients treated for FAI.	Retrospective chart review of patients who underwent HA for labral repair and FAI was performed with ≥ 12 months FU data.	Patients with IPT were matched at a 1:1 ratio to controls based on age, sex, and BMI. Demographic characteristics, radiographs and advanced imaging,	≥ 4/9	40 patients with IPT were identified and matched to 40 controls. Increased H was associated with increased IPT risk. For each 1-point JH score increase there was 1.69X (95% CI, 1.25-2.29; p < .001) increased odds of post-operative IPT. A high (>= 4) JH score was	Increased JH was associated with increased postoperative IPT after HA in patients treated for FAI and labral pathology.

			surgical characteristics, and cortico-steroid injection therapy data were obtained.		associated with an increased IPT likelihood (OR = 9.82; 95% CI, 2.79-34.58; p < .001).	
Naal FD, et al.[69]	Determined results of FAI surgery at ≥ 2-year FU by means of PROM outcome measures and failure rates; assessed JH prevalence in FAI patients and its effect on outcomes; and identified other risk factors associated with treatment failure.	232 consecutive patients (118 females; mean age, 36 years) with 244 hips treated for symptomatic FAI.	Pre- and post-operative PROM at a mean 3.7-year FU. Oxford Hip Score, UCLA Activity Scale, EuroQol-5 Dimension Index, and JH. Satisfaction questions defined subjective failure. Conversion to THR defined objective failure.	≥ 4/9	All PROM values (P < .001) improved from pre-operative values at FU: Oxford Hip Score: 33.8 to 42.4; UCLA Activity Scale: 6.3 to 7.3; EuroQol-5 Dimension Index: 0.58 to 0.80. Overall, 34% of patients scored ≥4 for JH, and 18% scored ≥ 6. 11 hips (4.7%) were converted to THR. 24 patients (10.3%) were deemed subjective failures. No predictive risk factors were identified for subjective failure. Tönnis grade (p < .001) predicted objective failure (OR = 13; 95% CI, 4-45). There was a weak inverse association (r = -0.16 to -0.30) between H score and pre-operative PROM values. There were no significant associations between JH score and post-operative PROM values or subjective failure rates, but patients who objectively failed had lower JH scores than did non-failures (1.6 vs 2.6; p = .049).	FAI surgery yielded favorable outcomes. H was not associated with subjective and objective results. Hip joint degeneration was the most important THR conversion risk factor. Although statistical significance was not reached, female patients with no joint degeneration, only mild FAI deformity, and higher Oxford scores at surgery seemed to be at increased subjective dis-satisfaction risk.
**Knee Joint-Specific or Arthroscopy JH**						
**Study**	**Purpose**	**Subjects/Methods**	**Tools**		**Results**	**Conclusion**
Astur DC, et al.[35]	Evaluated JH prevalence in patients traumatic meniscus and ACL injury surgery, and the influence of JH on post-operative results.	242 patients who underwent ACLR, partial meniscectomy, or combined ACLR and partial meniscectomy.	The TASS was used for postoperative assessment.	≥ 4/9	107 surgeries were performed to treat ACL injuries, 75 to treat ACL injuries with meniscus injuries, and 60 to treat meniscus injuries. Of the total, 45 patients had JH.	No association was found between JH and the evaluated variables (sex and type of specific injury). JH did not have a negative impact on post-operative outcomes.
Batty LM, et al.[37]	Investigated factors associated with a high-grade pre-operative pivot shift and evaluated the relationship between knee rotatory laxity and baseline PROM.	618 patients with ACL deficiency deemed high re-injury risk (18.9 ± 3.2 years of age). A binary logistic regression model was developed, with high-grade pivot shift as the dependent variable.	Age, sex, JH score, ACL injury chronicity, posterior third medial or lateral meniscal injury, and tibial slope were selected as independent variables. The importance of GR > 10° as a JH component was assessed using ROC curves. Baseline IKDC, ACL QOL, KOOS, or KOOS subscale scores were compared between patients with and without a high-grade pivot shift.	≥ 4/9 and GR ≥ 10°	6 factors were associated with a high-grade pivot shift: BSS score with each additional point; OR = 1.17; 95% CI, 1.06-1.30; p = 0.002), male gender OR = 2.3; 95% CI, 1.28-4.13; p = 0.005, presence of a posterior third medial OR = 2.55; 95% CI, 1.11-5.84; p = 0.03, or lateral OR = 1.76; 95% CI, 1.01-3.08; p = 0.048 meniscal injury, PTS > 9° OR = 2.35; 95% CI, 1.09-5.07; p = 0.03, and chronicity > 6 months OR = 1.70; 95% CI, 1.00-2.88; p = 0.049). GR presence improved JH score use as a high-grade pivot shift predictor. PTS < 9° was only associated with a high-grade pivot in the presence of a posterior third medial meniscal injury. Patients with a high-grade pivot shift had higher baseline 4-Item Pain Intensity Measure scores than did those without a high-grade pivot shift (mean ± SD, 11 ± 13 vs 8 ± 14; p = 0.04); however, there was no difference between groups for baseline	JH, male sex, posterior third medial or lateral meniscal injury, increased PTS, and chronicity were associated with a high-grade pivot shift in this population deemed high repeat ACL injury risk. The effect of PTS may be accentuated by the presence of meniscal injury, supporting the need for meniscal preservation. Baseline PROMs were similar between patients with and without a high-grade pivot shift.

					IKDC, ACL-QOL, KOOS, or KOOS subscale scores.	
Brinkman JC, et al.[38]	Compared surgical outcomes of HTA + LET vs. an all-soft-tissue QA without LET.	Compared high risk patients including those with a ≥ +2 pivot shift and H undergoing ACLR with either a HTA, HTA + LET and all soft tissue QA. No other procedures other than meniscal surgery.	IKDC and LKS scores were obtained pre-operatively, 3, 6, 12 and 24-month FU.	≥ 4/9	166 patients underwent ACLR. 63 patients in the HTA group, 47 in the HTA + LET group and 56 in the QA group. Mean age was 17.8 years. Mean H score was 3.2 with 24% of the population having JH. The overall rate of ≥ +2 pivot shift laxity was 82%. There were no group differences with regards to H% or 2+ pivot shift. All groups had more females than males (HTA group 66%; HTA + LET group 62% and QA group 56%). The QA group had a larger graft diameter (9.6 mm) compared to the HTA group (7.9 mm) and the HTA + LET group (8.1 mm)($p < .001$). The number of meniscal repair or partial meniscectomy procedures was similar between groups with 21.1% undergoing meniscectomy and 48% of the entire population undergoing meniscal repair. The study consisted of 68.4% athletes in the HTA group, 62.4% in the HTA + LET group and 72.6% in the QA group ($p =.32$). Both IKDC and LKS scores increased at each FU. There was no group difference at any time point for either IKDC or LKS scores. There was no group differences for contralateral ACL tears, arthrofibrosis or infection.	All-soft-tissue QA or the addition of an LET to a HTA both decreased graft re-tear rates and residual ≥ 2 pivot shifts compared to HTA graft alone. The use of QA + LET, however, did not improve PROM at 2-year FU compared to HTA alone. RTS was faster with QA compared to the HTA and HTA + LET; however, the addition of an LET to a HTA did not increase the RTS rate. High risk patients including those with JH and > ≥ grade 2 pivot shifts should undergo ACLR with either a QA or have a LET added to a HTA.
Feller JA, et al.[45]	Evaluated modified Ellison procedure use in patients deemed high risk for graft injury post- primary ACLR.	25 consecutive patients with at least 2 risk factors: age < 20 years at surgery, previous contralateral ACLR, family ACL rupture history, JH (BSS ≥ 4), grade +3 pivot shift on clinical exam, a desire to return to a pivoting sport, and an elite or professional status.	At FU patients completed the IKDC subjective knee evaluation, KOOS-QOL subscale, ACL-RIS, Marx Activity Rating Scale, and SANE score.	≥ 4/9	At 12-month FU the mean outcome scores were: SANE, 94/100; IKDC, 92/100; Marx, 13/16; ACL-RIS, 85/100; and KOOS, 77/100. At 24 month FU, RTS data were available for 23/25 patients; 17 (74%) were playing at the same level or higher than pre-injury and 2 at a lower level. One patient (4%) sustained a contact mechanism graft rupture at 12 months. There were 2 (9%) contralateral ACL injuries, including 1 ACL graft rupture, at 11 and 22 months post-operatively. One experienced a contralateral ACL graft rupture at 26 months.	Modified Ellison procedure use for LET augmentation of primary ACLR led to a low graft rupture rate suggesting efficacy for graft injury prevention.
Getgood AM, et al.[48]	Evaluated if adding LET to HTA ACLR would reduce the clinical failure rate of young patients with an ACL deficient knee and high re-injury risk.	618 patients (mean age = 19 years, range = 14-25 years, 51% women) with an ACL-deficient knee with high re-injury risk. All underwent standardized, single bundle HTA ACLR. 306 patients were randomized to ACLR + modified Lemaire LET using a iliotibial band; the other patients underwent HTA ACLR alone (n = 312).	High re-injury risk was defined as: ≥ 2 of competitive pivoting-sport participation, ≥ grade 2 pivot-shift, JH score ≥ 4, or GR >10°.	≥ 4/9, or GR >10°.	ACLR with LET reduced clinical failure and graft rupture at 2 years compared with ACLR alone. Pain was less in the ACLR-alone group vs. ACLR + LET at 3 months ($p = 0.003$). ACLR alone had a greater improvement vs. ACLR + LET for IKDC and KOOS domain scores at 3, 6 months ($p < 0.05$). At 2 year FU groups did not differ for any PROM, including the Marx Activity Rating Scale, ($p > 0.10$).	Adding a modified Lemaire LET to single-bundle HTA ACLR in young patients with an ACL-deficient knee at high risk for re-injury, reduced clinical failure rate at 2 year FU.
Helito CP, et al.[51]	Compared functional outcomes, residual	Both groups consisted of patients with ACL	Group 1 patients had anatomical HTA	≥ 5/9	90 patients with JH who underwent ACLR were evaluated.	Combined HTA ACLR and ALL

	instability, and rupture rates in patients with JH undergoing isolated HTA ACLR or combined HTA ACLR and anterolateral ligament (ALL) reconstruction.	injuries and associated JH.	ACLR, and group 2 patients had anatomical HTA ACLR with ALL reconstruction. Group 1 consisted of historical controls. Associated meniscal injury, subjective IKDC and LKS scores, KT-1000 measurements, residual pivot-shift, and graft rupture rates were evaluated.		Mean FU was 29.6 ± 6.2 months for group 1 and 28.1 ± 4.2 months for group 2 (p = .51). Group differences were not observed for JH, gender, injury duration before ACLR, FU, pre-operative instability, or associated meniscal injuries. Mean age was 29.9 ± 8.1 years in group 1 and 27.0 ± 9.1 years in group 2 (p = .017). In the final evaluation, group 2 had better AP stability (p = .02), better pivot shift rotational stability (p = .03) and a lower ACLR failure rate (21.7% [group 1] vs 3.3% [group 2]; p = .03). Post-surgical functional scales showed no group differences for IKDC (p = .27) or LKS (p = .41).	reconstruction in patients with JH resulted in a lower failure rate and improved knee stability compared to isolated HTA ACLR.
Hosseinzadeh N, et al.[52]	Compared the outcomes of quadruple HTA ACLR between JH and NJH patients.	36 patients with JH underwent HTA ACLR. 44 matched NH patients were included in the control group. Mean FU was 20.65 ± 6.93 months.	The outcomes were evaluated using the Lachman test, pivot shift test, anterior tibial translation and KT-1000 side-to-side difference, and IKDC score.	≥ 4/8 (maximum of 8 rather than 9)	Lachman and pivot shift test results did not differ between JH and NH patients (p = 0.67 and p = 0.27, respectively). Mean ATT was 7.06 ± 1.41 mm in the JH group and 6.11 ± 1.53 mm in the NH group (p = 0.006). Mean KT-1000 side-to-side difference was 2.25 ± 1.31 mm in the patient group and 2.5 ± 1.44 mm in the control group (p = 0.42). Mean IKDC scores did not differ between groups (66.1 ± 20.6 vs. 69.9 ± 16.1, p = 0.35). ACLR failure occurred in 2 (5.5%) patients of the JH group and no patients of the control group (p = 0.21).	ACLR with quadruple HTA was an effective treatment for JH patients, at short-term FU.
Juul-Kristensen B, et al.[55]	Evaluated knee function in children and adults with JH and NJH.	39 children and 36 adults (mean age children 10.2 years; adults 40.3 years) were included, comprising 19 children and 18 adults with H.	Knee function was determined by PROM for physical fitness (10 cm VAS), KOOS) (only adults), peak isokinetic knee strength (60°/s) and peak vertical jump displacement (PVJD), with calculated knee strength balance, hamstring/quadricep s (H/Q) strength ratio and peak rate of force development.	≥ 5/9 for children; ≥ 4/9 for adults; and with GR at one knee	Regardless of age and knee pain adults with JH had lower knee function scores (KOOS: pain, p = 0.001; symptoms, p = 0.001; ADL p = 0.001; Sport/ Recreation, p = 0.003; knee-related quality of life, p < 0.001), and hamstring/ quadriceps ratio (0.46 vs. 0.54, p = 0.046) than adults with NJH.	Children at 10 years with JH had normal, but adults with H had impaired knee function. Children with JH should be followed longitudinally. Greater attention to knee function should be given to children with JH who have parents with JH.
Keizer MNJ, et al.[57]	Determined if patients who had undergone HTA ACLR compensated for ATTd by developing coper neuromuscular activation strategies.	40 patients who underwent unilateral HTA ACLR performed 10 single-leg hops for distance with both legs.	Lower body kinematic and kinetic data were measured using a motion-capture system, and ATTd was determined. Muscle activity was measured with sEMG. Bilateral ATTd was measured using a KT-1000 arthrometer.	0-9/9	There was no correlation between ATTp and ATTd in copers; however, there was a positive correlation between ATTp and ATTd in the surgical knee of noncopers. There was a positive correlation between JH score and ATTp and between JH score and ATTd for the surgical knee in both copers and noncopers. Copers showed a negative correlation between surgical knee ATTd and gastrocnemius activity during landing. Noncopers showed a + correlation between surgical knee ATTd and knee flexion moment	Copers used different landing techniques than noncopers. To reduce ATTd, copers used increased gastrocnemius activity, whereas noncopers moderated ATTd by generating a smaller knee flexion moment. Patients who RTS after HTA ACLR had sufficient plantar flexor activation to limit ATTd.

					during hop landing.	
Kim SJ, et al.[59]	Compared 2- and 5-year outcomes of BPTB or HTA ACLR between patients with and without JH and compared the 2 graft types in patients with H.	237 patients who underwent ACLR were included. Patients were classified into JH or NJH groups, and further subdivided based on graft type.	Stability was measured using Lachman test, pivot-shift test, and ATT (KT-2000). Functional outcomes were measured using the LKS, and IKDC subjective score. IKDC objective grade and radiographic grade were also assessed. Clinical assessments were conducted pre-operatively and at 2 and 5 year FU.	≥ 4/9	Patients with JH receiving HTA had poorer outcomes than those without JH at 2-year FU. At 5-year FU patients with JH had poorer outcomes than patients without JH irrespective of graft type. In patients with JH the BPTB graft provided better stability and functional outcomes than the HTA at both 2- and 5-year FU. Comparisons between serial outcomes measured at 2 and 5 years revealed that stability and functional outcomes deteriorated over time in patients with JH.	Less satisfactory stability and functional outcomes were noted in patients with JH compared to patients without JH. Comparisons of stability and functional outcomes after ACLR in patients with JH between two different grafts revealed that the BPTB graft achieved better results than the HTA.
Larson CM, et al.[62]	Evaluated whether JH and contralateral knee GR affected failure rates and PROM after ACLR.	226 consecutive patients with acute ACL tears were evaluated for JH. Minimum 2-year FU was achieved for 183 knees (81%). Patients underwent ACLR with either BPTB autograft (n = 46), quadrupled HTA (n = 85), or tibialis anterior allograft (n = 52).	ATT (KT-1000), IKDC, Cincinnati knee score , and LKS scores were obtained.	3-4/9	41/183 consecutive patients were categorized as JH. At mean 6 year FU. IKDC (p = .003), Cincinnati knee score (p = .001), and LKS scores (p < .001) were better in the NJH group for patients with an intact graft. The failure rate was higher in the JH group (10 knees, 24.4% failure rate) compared with the NJH group (11 knees, 7.7% failure rate) (p = .006). Overall ACL injury rate (ACL graft injury, excessive graft laxity, plus contralateral ACL tear) was higher in the JH group (34.1%) compared with the NJH group (12%) (p = .002). Heel height > 5 cm (p = .009) and fifth MCP extension > 90° (p = .006) were independently predictive of failure.	Graft failure rates were higher and inferior subjective outcomes were observed after ACLR in patients with JH. Heel height and fifth MCP hyperextension were most predictive of ACL injury/ re-injury and poorer outcome scores. Nearly 1/3rd of H patients sustained a contralateral ACL tear, ipsilateral graft failure, or had excessive graft laxity.
Lindskog J, et al.[64]	Studied differences in pre-injury level of activity, muscle function and PROM at the time of RTS or RTP, as well as the time of RTS or RTP in patients with JH compared with NH patients over the first 2 years post-ACLR.	Data was obtained from an ACL- and rehabilitation-specific register. Patients between 16-50 years of age who had a primary ACLR were included. Data up to 2 years post-ACLR were used.	RTS and RTP, results from isokinetic muscle function tests for knee extensors and flexors and PROM: Knee Self-Efficacy Scale, KOOS Score and ACL-RTS after Injury scale at the time of RTS, and RTP. A TASS ≥ 6 was used to define RTS, while a TASS ≥ pre-injury level was used to define RTP.	≥ 5/9	1,198 patients (54.7% women) with a mean age of 28.5 ± 8.6 years were included. Less patients with JH achieved RTS than NH patients (49.2% vs. 57.3%, OR = 0.72, p = 0.041). Patients with JH were less symmetrical on the knee extension strength test (LSI), at RTP compared with NJH patients (87.3% ± 13.5 vs. 91.7% ± 14.3, Cohen's d = 0.142, p = 0.022).	Fewer patients with JH achieved RTS compared with NJH patients. Patients with JH had less symmetrical knee extensor strength at RTP compared with NJH patients.
Lodhia P. et al.[65]	Investigated clinical outcome differences between 4S and 5S HTA for ACLR in patients who underwent ACLR alone or with an LET procedure.	Data from the STABILITY study were analyzed to compare a patient sub-group undergoing ACLR alone or ACLR + LET with a minimum graft diameter of 8 mm using either a 4S or 5S HTA.	The primary outcome was clinical failure (composite of rotatory laxity and/or graft failure). Secondary outcomes consisted of the ACL-QoL and the IKDC score at 24 month FU.	0-9/9	Of 618 patients randomized in the study, 399 (228 male; 57%) fit the inclusion criteria. Of these, 191 and 208 patients underwent 4S and 5S HTA ACLR, respectively, with a minimum 8-mm graft diameter. Groups were similar other than for anthropometric factor differences: sex, height, weight, and JH. The primary outcomes revealed no group differences for rotatory stability (OR = 1.19; 95% CI, 0.77-1.84; p = .42) or graft failure (OR = 1.13; 95% CI, 0.51-2.50; p = .76). There was also no group difference for	At 24-month FU there were no clinical failure rate or PROM differences in patients with 4S and 5S HTA of ≥ 8-mm diameter for ACLR or ACLR + LET. The 5S HTA configuration was proposed as a viable option for a larger-diameter ACL graft.

					Lachman (p = .46) and pivot-shift (p = .53) test results at 24 month FU. Secondary outcomes revealed no group differences for ACL-QoL (p = .67) and IKDC (p = .83) scores.	
Marmura H, et al.[67]	Attempted to determine if the KOOS had adequate structural validity for use with young, active patients with ACL tears.	In randomized controlled multi-center study, 1033 patients were screened for eligibility. Patients were eligible if they had an ACL deficient knee, were 14-25 years of age, and were considered high re-injury risk.	Higher ACLR re-injury risk was based on ≥ 2 of the following factors: pivoting sport participation, ≥ + 2 grade pivot shift, and JH. In total, 618 patients were randomized into the trial.	≥ 4/9 or GR > 10°	98% (605/618) of patients participated. A cross sectional retrospective secondary data analysis of the Stability 1 baseline KOOS data was completed using exploratory, confirmatory factor analyses to assess the validity of the 5-factor KOOS structure (symptoms [seven items], pain [nine items], ADL [17 items], sport and recreation [five items], and QOL [four items]) for young active patients with ACL tears.	The five-factor KOOS structure was not valid in this sample of young, active patients undergoing ACLR.
Parmar R, et al.[71]	Evaluated the efficacy of adding LET to ACLR in competitive female soccer athletes with greater preoperative JH.	Reviewed female high school and collegiate soccer players who underwent primary ACLR with at least 2-year FU.	Participants were divided into 2 groups: those who received ACLR alone (BPTB, QA or HTA) and those who received ACLR + LET. Patient demographics and physical exam findings, including pivot shift results, were collected. Positive pivot-shift referred to a grade ≥2. Patient outcomes included graft failure (defined as ACL re-tear), IKDC score, LKS, RTS, and complications were evaluated.	≥ 4/9	133 players who underwent ACLR met inclusion criteria, including 43 that received an ACLR + LET and 90 patients who underwent isolated ACLR. Average FU was 39.0 and 36.1 months in the LET group and the control group, respectively. Patients who underwent LET had a greater H rate (48.8% vs 18.9%; p < .001) and greater median JH score (3.0 vs 1.0; p < .001) than those without LET. There was a similar graft failure rate in both groups (4.7% vs 3.0%; p = .658). On the basis of MCID thresholds, there were no clinically relevant group differences in IKDC, LKS scores. RTS rates were also comparable between the LET and control cohorts (90.7% vs 85.6%; p = .81).	The addition of LET to ACLR in female soccer players with pre-operative JH yielded graft re-tear and RTS rates comparable to those of NJH athletes.
Pfeiffer TR, et al.[72]	Determined which factors, including sex, were associated with increased rotatory knee laxity.	98 college athletes (median age = 20, range = 18-25 years) with no knee injury history.	IKDC and Marx activity scores were obtained and subjects underwent ATT and knee hyperextension measurements bilaterally. A standardized pivot shift test was performed in both knees and quantified using image analysis technology.	≥ 5/9	Average lateral compartment ATT during the pivot shift test was 1.6 mm (range = 0.1-7.1) with a mean side-to-side difference of 0.6 mm (range = 0-2.7). The average ATT with the Lachman test was 9 mm (range = 2-15). Lateral compartment ATT during the pivot shift test was higher in females (median, 1.6 mm; range = 0.3-4.9 mm) than in males (1.1 mm. range = 0.1-7.1 mm) p < 0.05). Lateral compartment ATT during the pivot shift test was correlated with ATT during the Lachman test (r = 0.34; p < 0.05). There was no significant correlation between lateral tibial ATT during the pivot shift test and knee hyperextension or JH.	Female sex was associated with increased rotatory knee laxity measured during the pivot shift test and ATT during the Lachman test.
Sahin S, et al.[75]	Studied the relationship between JH scores and biomechanical risk factors, such as knee valgus.	25 adolescent, club-level female volleyball players (14.5 ± 1.8 years of age).	Peak coronal plane angles were computed during DLVJ, SLS, and SLDL. Spearman correlations were performed to identify relationships between JH scores	0-9/9	Peak knee valgus was moderately correlated with JH score during the DLVJ-Landing (r = 0.487, p = 0.014), SLDL (r = 0.478, p = 0.016), and SLS (r = 0.439, p = 0.028).	Adolescent volleyball players with higher JH scores had greater peak knee valgus, suggesting that they might benefit from a targeted neuro-muscular training or injury prevention

			and biomechanical variables.			programs.
Sundemo D, Blom A, et al.[80]	Studied whether increased rotatory knee laxity was associated with greater JH in ACL-injured and contralateral knees.	103 patients across 4 international centers who underwent anatomic ACLR. Rotatory knee laxity was evaluated using standardized pivot shift testing pre-operatively (while conscious and under anesthesia).	Joint acceleration was measured using an inertial sensor. An image analysis system was used to measure lateral compartment ATT during pivot shift testing. The correlation between H and the rotatory knee laxity was determined bilaterally.	≥ 5/9	96 patients (83 and 13 in the NJH and JH groups, respectively). There was a relationship between H score and pivot shift magnitude at the non-involved knee (r = 0.235, p < 0.05). When analyzing the same knee, multivariate analysis adjusted for meniscal injury, age and gender revealed an increased OR for patients with increased lateral compartment ATT to be part of the high JH score group (OR = 1.86, 95% CI 1.10-3.17, p < 0.05).	There was a weak correlation between JH and the contralateral healthy knee, indicating increased rotatory knee laxity. JH did not correlate with rotatory knee laxity in ACL-injured knees.
Sundemo D, et al.[81]	Evaluated whether JH influenced post-operative results, including RTS (PROM, hop tests, muscular strength, and ACL re-injury rates, in patients 1 year post=ACLR.	Data was extracted from a rehabilitation-registry. Patients 16-50 years of age previously undergoing ACLR with available 1 year FU data were eligible.	Patients were examined 1 year post-ACLR for RTS, PROM, hop tests, muscular strength and re-injury rate. Patients were allocated into JH and NH groups. The KOOS sports and recreation subscale was the primary PROM.	≥ 5/9	356 patients (41% males) were included, of which 76 (24% male) had JH. Patients with JH had inferior knee extensor pre-operatively (mean 81.6% vs. 91.4%, p = 0.02) and knee flexor (mean 91.9% vs. 99.1%, p = 0.047) LSI strength compared to NH patients. There were no group differences for KOOS sports and recreation subscale scores, nor for any other post-operative outcomes. 9 patients (11.8%) in the JH group suffered ACL re-injury, compared to 13 control patients (4.6%)(n.s.).	At 1 year post-ACLR JH did not affect post-operative patient satisfaction, strength or functional outcome. No conclusive statements could be made regarding the influence of JH on ACL re-injury risk.
Vaishya R, Hasija R.[83]	Compared JH rates in patients with and without ACL injury.	135 men and 75 women (mean 24.6 years of age) who underwent ACLR were reviewed and compared with 55 male and 35 female controls with no knee injury.	JH was evaluated by 2 examiners.	≥ 4/9	127 (60.5%) of patients with ACL injury and 23 (25.5%) of controls had JH (p < 0.01). Among them, 58% and 24% were men and 65% and 29% were women, respectively. Female sex was associated with JH. Patients with ACL injury were more likely to have JH (OR = 4.46).	JH was more common in patients with ACL injury.
Westin M, et al.[87]	Evaluated possible intrinsic risk factors for ACL re-injury in competitive alpine skiers.	384 (191 males/193 females) from a Swedish ski high school were followed. 31 (5 males/26 females) had undergone ACLR before school entry. Ipsilateral re-injury and contralateral injury risk factors were analyzed.	Clinical and physical exam, SF-36 and injury rates were compared	≥ 5/9	12/31 students (39%), 10 female and 2 male skiers, 16.5 ± 0.5 years of age, sustained ACL re-injury. 10/12 ACL re-injuries occurred within 10-23 months after the first injury (14.8 ± 4.7 months) and two ACL re-injuries occurred at 29 and 47 months, respectively, after the first injury. 8 ACL re-injuries were at the ipsilateral knee and 4 were at the contralateral knee. There were no group differences for LE muscle flexibility, JH score, single leg hop for distance or square hop test performance. Side-to-side differences were found with respect to knee joint laxity > 3 mm (KT 1000), (p = 0.02), and the side hop test (p = 0.04).	Side-to-side hop test and knee joint laxity may predispose ACL graft re-injury.
Zhang ZY, et al.[88]	Described pre-operative ATS and RTS in ACL-injured and ACL-intact knees and identified related factors for ATS and RTS based on MRI images.	Demographic data and pre-operative MRI of 104 ACL-injured patients were analyzed. ACL-intact knees were 1:1 matched as the control group.	ATS was measured using based on longitudinal tibial axis alignment, and RTS was determined by the difference between lateral and medial ATS. Related factors for ATS and	0-9/9	Increased lateral ATS (p < 0.0001), medial ATS (p < 0.0001) and RTS (p = 0.0479) were observed in ACL-injured knees compared with the control group. Increased PTS, JH score ≥ 4, meniscal injury and long injury-to-MRI time were correlated with an ATS increase. Factors	In ACL-injured knees, the tibia subluxated anteriorly in both lateral and medial compartments, and also internally rotated. During pre-surgical planning attention should be placed on

			RTS were examined.		contributing to increased RTS were increased lateral PTS, JH score ≥ 4, lateral meniscal injury, and left side.	factors associated with altered tibiofemoral position.
Ziegler CG, et al.[90]	Assessed patient history and physical exam findings comparing patients with ACL graft failure compared with primary ACL tear patients to better discern risk factors for ACL graft failure.	Compared patients who underwent revision ACLR (BPTB autograft, HTA, QA, BPTB allograft, or HTA) with a primary ACLR group (BPTB autograft, HTA, or BPTB allograft). Preoperative history, exam, and imaging data were collected.	MRI, x-rays, and 3D CT. Patients had undergone primary ACLR by a single surgeon at a single center with minimum 1-year FU or ACL graft failure with revision ACLR performed by the same surgeon.	0-9/9	109 primary ACLR patients, mean age 33.7 years (range 15-71), and 90 revision ACLR patients, mean age 32.9 years (range 16-65), were included. The revision ACLR group had increased mean JH scores (4 vs. 0; p < .001) and greater side-to-side quadricep circumference differences (2 cm vs. 0 cm; p < .001) compared with the primary ACLR group. ACL family history was more likely in the revision group (47.8% vs. 16.5%; p < .001). The revision group had an increased lateral PTS (7.9° vs. 6.2°), antero-lateral tibial subluxation (7.1 mm vs. 4.9 mm), and antero-medial tibia subluxation (2.7 mm vs. 0.5 mm; all p < .005). In the revision group, femoral tunnel malposition occurred in 66.7% in the deep-shallow position and 33.3% in the high-low position. Tibial tunnel malposition was 9.7% from medial-to-lateral and 54.2% from anterior-to-posterior. 56 patients (77.8%) had tunnel malposition in ≥ 2 positions. Allograft tissue was used for the index ACLR in 28% in the revision group compared with 14.7% in the primary group.	BSS score, side-to-side quadriceps circumference difference, family ACL tear history, lateral PTS, anterolateral tibial subluxation, and anteromedial tibia subluxation were each different between primary and revision ACLR groups. In addition, there was a high rate of tunnel malposition in the revision ACLR group.
Zsidai B, et al.[9]	Determined the 12-month risk of a second ACL injury in JH and NJH patients who RTS at competition level post-ACLR	153 patients, 50 (22.2%) with JH and 175 (77.8%) NH. Data were extracted from a rehabilitation- registry for 16-50-year-old patients treated with ACLR.	Demographics, outcome data and the second ACL injury incidence ≤ 12 months post- RTS, defined as a new ipsilateral or contralateral ACL, were compared between JH and NJH patients.	≥ 5/9	Within 12 months of RTS, 7 (14%) patients with JH and 5 (2.9%) NJH had a second ACL injury (p=0.012). The odds of sustaining a second ipsilateral or contralateral ACL injury were 5.53X (95% CI 1.67-18.29) higher in patients with JH compared with NJH patients (p = 0.014). The lifetime HR of a second ACL injury post-RTS was 4.24 (95% CI 2.05-8.80; p = 0.0001) in patients with JH. No between-group PROM differences were observed.	Post-ACLR patients with JH had over 5X greater odds of sustaining a second ACL injury after RTS. The importance of joint laxity assessment should be emphasized in patients who aim to RTS post-ACLR.
**Ankle Joint-Specific or Arthroscopy JH**						
**Study**	**Purpose**	**Subjects/Methods**	**Tools**		**Results**	**Conclusion**
Baillie P, et al.[36]	Compared clinical exam findings between elite athletic populations with and without PAIS.	10 male and female professional ballet dancers and athletes with PAIS were matched for age, sex, and activity to 10 professional ballet dancers and athletes without PAIS.	SLHR endurance test, ankle dorsiflexion, ankle plantar flexion, and first MTPJ dorsiflexion ROM, and CAIT questionnaire completion.	≥ 5/9	The PAIS group performed fewer SLHR repetitions (p = 0.02) and were more symptomatic for perceived ankle instability according to CAIT scores (p = 0.004). Only 1 subject was positive for H score.	SLHR endurance capacity was lower, and perceived ankle instability was greater in the PAIS group.
Hou ZC, et al.[53]	Compared balance training outcomes in patients with CAI who were JH or NJH.	40 patients with CAI were assigned into JH (n = 20) and NJH (n = 20) groups receiving the same 3-month balance training.	FAAM, number of patients experiencing ankle sprain, isokinetic muscle strength and postural control tests were analyzed.	≥ 4/9	Groups did not differ at baseline except for the JH group having poorer posteromedial (83.6 ± 10.1% vs. 92.8 ± 12.3%) and posterolateral 84.7 ± 11.7% vs. 95.7 ± 8.7%) SEBT test results. Post-balance training the	Patients with CAI and JH had equal or better postural stability and muscle strength improvements post-balance training compared to NJH

					JH group had a lower re-sprain ratio immediately (11.1% vs. 23.5%), and 3 months (16.7% vs. 29.4%) post-training, than the NJH group, greater FAAM Sport scores, and greater plantar flexor and dorsiflexor strength. Compared with baseline, groups had similar FAAM ADL scores, muscle strength and balance control (SEBT in the posterior-lateral and posterior-medial directions), and BESS scores.	patients. Balance training might be effective for CAI patients with JH before considering surgery.
Porter MD, et al.[73] .	Attempted to determine if clinical outcomes were similar in patients with BW > 90 kg or JH, relative to controls.	66 patients were divided into 3 groups: controls (no risk factors for inferior clinical outcomes), patients with BW > 90 kg, and patients with JH. All underwent LCL complex imbrication with LARS augmentation.	TASS and FAOS subscale scores, ankle instability recurrence, need for further surgery, and/or complications. Group outcomes were compared at 2- and 5-year FU.	≥ 5/9	21 patients/group). TASS improved from pre-operative values in all groups by 2- and 5-years FU (p <.001 for all). Relative to controls, TASS scores were lower in the > 90-kg group at 2- and 5-years (p <.001), while the JH group had similar scores to controls at both FU periods. Both the > 90-kg and the JH groups showed no significant improvement difference on any FAOS subscale score relative to controls, at both 2- and 5-year FU. There were no recurrences, repeat surgeries, or major complications.	Compared to controls, patients with BW > 90 kg or JH had similar FAOS. TASS were lower in the > 90-kg group, at 2- and 5-years. LARS was a viable option in patients for whom the MBG procedure is relatively contraindicated.
Wang A, et al.[86]	Compared clinical outcomes and RTS between anatomic reconstruction with gracilis autografts and the MBG procedure in patients with JH.	19 patients underwent anatomic reconstruction, and 49 underwent the MBG procedure.	FAOS, Karlsson score, RTS, ROM	≥ 4/9	Mean FU was 38.3 months in the reconstruction group and 43.7 months in the MBG group. The FAOS and Karlsson scores improved post-surgery in both groups (p <.001), with the reconstruction group having higher post-operative FAOS-Sports scores (87.9 ± 8.9 vs 80.5 ± 11.6; P =.015) and Karlsson scores (86.9 ± 6.1 vs 82 ± 8.4; p =.025) than the MBG group. The RTS rate was higher in the reconstruction group than in the MBG group (73.3% vs 38.9%; p =.034). The MBG group also had a higher sprain recurrence rate (22.4% vs 0%; p = .027). More patients reported dorsiflexion restriction in the reconstruction group (n = 4; 21.1%) than in the MBG group (n = 1; 2%) (p =.019); there was no noticeable effect on daily life and sports.	Better clinical outcomes, less sprain recurrence, and a higher RTS rate were found after anatomic reconstruction with free tendons compared with the MBG procedure in patients with JH.
