# Supplementary material for: Beighton Scoring System Use in Generalized Joint Hypermobility Studies Has Greater Scientific Rigor Than Joint‐Specific or Arthroscopy Joint Hypermobility Studies
Source: Arthrosc Sports Med Rehabil. 2026 May 4;8(2):e70000. doi: 10.1002/ars2.70000 (PMC13307201; doi:10.1002/ars2.70000)
Supplement: Supplementary file 1 — Supplementary Material [file ARS2-8-e70000-s001.zip › ASMAR_SUPPLEMENTAL_TABLE_1z.pdf]

Supplemental Table 1. General JH study publication source, study purpose, results, and conclusion. ACL = anterior cruciate ligament; AROM = active range of motion; BMI = body mass index; BSS = Beighton Scoring System; CCI = co-contraction index; ER = external rotation; FPI = foot posture index; JH = joint hypermobility; HQ = hamstrings, quadriceps; HRQoL = health related quality of life; LE = lower extremity; LLAS = Lower Limb Assessment Scale; MS = musculoskeletal; n.s. = not significant; NCAA = national collegiate athletic association; RTS = return to sports.

Study	Purpose	Results	Conclusion
Armstrong R, Greig M.[34]	Studied rugby players, netball players, dancers and control subjects for influence of H on injuries	BSS scores were predictive of arthralgia and joint dislocation or subluxation.	BSS score information might enable clinicians to implement more effective injury prevention strategies
Bronner S, Bauer NG.[39]	Examined MS injury risk factors of dancers based on JH	Dancers with low and high BSS scores were 1.43X and 1.22X times more likely to sustain injury than dancers with mid-range scores ($p \leq 0.03$). Dancers with better technique (low-medium BSS scores) were 0.86X and 0.63X less likely to sustain injury ($p = 0.013$ and $p < 0.001$) compared to those with poor technique. Dancers with one, or 2-4 tight muscles were 2.7X and 4.0X more likely to sustain injury ($p \leq 0.046$). Dancers with 2-4 injuries in the previous year were 1.38X more likely to sustain subsequent injury ($p < 0.001$).	Dancers with either hypo- or JH risk factors might benefit from injury prevention programs
Bukva B, et al.[40]	Evaluated the relationship between artistic gymnast JH and injury rate over a 1-year period	The most common injury was low back pain, followed by knee, shoulder, hip and ankle injuries. There was a strong correlation between gymnastics training years and injury rate ($P < 0.001$).	Training hours/week, not BSS score related to injury rate
Clinch J, et al.[43]	Evaluated the prevalence and pattern of JH among children	JH prevalence in girls and boys was 27.5% and 10.6%, respectively. 45% of girls and 29% of boys had finger H. There was a positive association between JH in girls and physical activity, BMI, and maternal education. No associations were identified in boys.	BSS score ≥ 4 threshold might be inappropriately low in subjects with a developing MS system.
Collinge R, Simmonds JV[2]	Evaluated the influence of JH on football player injuries over the season.	Similar injury rates were observed among H and non-H athletes. Once injured, however, the H group missed more training days or games	To minimize re-injury risk, the RTS time periods for individuals with JH might be extended.
Decoster LC, et al.[44]	Studied JH among adolescent athletes during pre-season physical exam	More females (22%) than males (6%) had JH	Prospective studies were suggested to confirm JH injury risk vs. regular or strenuous exercise benefits
Frisch A, et al.[47]	Studied adolescent male soccer players during preseason examinations and injury incidence over the ensuing season	Failed to identify significant injury prevalence differences between players with H and those without.	A single pre-season JH examination may be insufficient in for injury prevention strategy development
Hanzikova I, et al.[49]	To better understand non-contact ACL injuries, evaluated the influence JH.	The JH group had lower minimum knee valgus angles with a mean difference of 3.5° ($P = 0.03$, Hedge $g = 0.69$) and greater peak knee ER with a mean difference of −4.5° ($P = 0.04$, Hedge $g = 0.70$) during dominant leg cutting, and lower peak ankle plantar flexion angles with a mean difference of 4.5°($P = 0.03$, Hedge $g = 0.73$) during nondominant leg cutting compared with the non-JH group.	Despite group cutting kinematic differences that were identified, based on existing evidence isolated kinematic variables were not deemed crucial to non-contact knee and ACL injury mechanisms
Hawke F, et al.[50]	To better understand the complex relationships between foot posture, flexibility and bone mass in children.	Higher FPI was associated with higher BSS score ($r = 0.44$, $p = 0.01$); greater lunge angle was associated with higher BSS score ($r = 0.40$, $p = 0.02$) and LLAS ($r = 0.42$, $p = 0.02$) scores; older age was associated with higher	Children with a more pronated foot had greater lower limb and whole-body flexibility, but not greater ankle joint flexibility.

		BMI (r = 0.52, p = < 0.01, and with lower BSS score (r = −0.41, p = 0.024), with lower LLAS (r = −0.40, p = 0.03) scores; and higher BSS score was associated with higher LLAS (r = 0.85, p = <0.01).	There was strong agreement between lower-limb and whole-body flexibility.
Johnson A, et al.[54]	Evaluated male professional soccer athletes for JH	Reported a strong relationship between LLAS and BSS scores (r = 0.73)	LLAS was a valid test for identifying lower limb JH
Juul-Kristensen B, et al.[56]	Studied the influence of JH among adolescent girl school children	Girls with JH had a lower lateral HQ CCI and a higher medial/lateral HQ CCI ratio and larger postural sway length during balance tasks.	Girls with JH and at least one JH knee performed static balance tasks with greater medial knee muscle activity relative to lateral activity and greater lateral postural sway when vision was eliminated.
Konopinski M, et al.[60]	Compared injury incidence between elite soccer players with or without JH	Players with JH had a higher injury incidence (mean [95% CI] difference, 5.2X [0.9-2.7] injuries/1000 h; p = 0.06). Training exposure increased injury risk (p < 0.001). Players without JH had lower injury risk (p = 0.11).	JH and training exposure both trended towards increased injury risk.
Krivickas L & Feinberg J[61]	A flexibility and injury incidence study of college athletes.	For each additional BSS point injury risk decreased 16%, however, for each additional point on the 10-point muscle tightness scale, injury risk increased 23%. Women had greater mean (+/-SD) laxity scores than men (3.3 +/- 2.2 vs 1.8 +/- 2.0; p < .001) and lower mean overall muscle tightness scores (1.5 +/- 1.6 vs. 3.5 +/- 2.1; p < .001). Among females, LE injury rate was unrelated to JH or to flexibility. Among men LE injuries were associated with lower JH scores (p = .008) and greater muscle tightness (p = .04).	Tight ligaments and muscles were related to injury in male, but not to female college athletes. A pre-season flexibility program may decrease injuries in male college athletes.
Nicolay RW, et al.[70]	Studied the influence of JH as a risk factor for injury in NCAA Division I football players	Based on treatment, and surgery histories a preseason JH diagnosis did not increase injury risk over the 2-year study period.	No specific pre-participation risk counseling or intervention was warranted for football players with JH.
Rejeb A, et al.[74]	Studied JH and injury rates among Middle Eastern youth athletes	BSS scores were not associated with injury in the overall cohort, however, they were associated with greater contact sport injury risk.	As an injury pre-emptive measure, preseason JH determination should be considered specifically for contact sports
Russek L & Errico D[23]	Evaluated prevalence of generalized JH and joint-specific JH and their relationship to musculoskeletal injuries and symptoms.	JH prevalence was 26.2% (females 36.7%, males 13.7%). Group injury rates did not differ. Individuals with JH joints were more likely to experience sprains, back pain and stress fractures. Individuals with joint-specific JH were also more likely to report clumsiness, easy bruising, and balance problems,	Although generalized JH was not associated with increased injury incidence or symptoms, joint-specific JH often was.
Scheper M, et al.[24]	Evaluated the effects of JH in professional dancers and a control group on physical fitness, musculoskeletal complaints, and psychological distress.	Overall, subjects with JH had decreased functional walking distance, had lower strength, had greater fatigue and had greater psychological distress. Compared to controls, dancers has lower BMI, greater walking capacity, were more fatigued and experienced more psychological distress.	Despite training, generalized JH was associated with lower physical fitness. Dancers with JH were more vulnerable to musculoskeletal and psychological complaints
Scheper M, et al.[22]	Attempted to establish the association between JH and functional status and to the contribution of physical fitness and musculoskeletal complaints to this association.	Generalized JH was negatively associated with decreased walking distance and jumping capacity compared to subjects without generalized JH.	Generalized JH was independently associated with lower walking and jumping capacity.
Schmidt H, et al.[76]	Compared musculoskeletal injury rates and postural sway in elite adolescent athletes.	LE function, injury prevalence, related factors (exacerbation, recurrence, and absence from training), HRQoL, and hop test distances did not differ for those	Suggested that ankle injury risk of subjects with JH should be studied in future longitudinal

		with and without JH. The JH group had greater sway test center-of-pressure path lengths.	studies.
Skwiot M, et al.[77]	Assessed the prevalence of JH using different criteria and injury risk factors among jazz dancers	JH prevalence differed depending on which criteria were adopted (p = 0.001) with the BSS, Grahame & Hakim questionnaire, and Sachse's criteria identifying 64.9%, 74% and 59.7% of the sample as JH, respectively. JH was more prevalent in women than men.	There is a need for more unified diagnostic criteria.
Smith R, et al.[78]	Evaluated the incidence of JH in young female netball players in relationship to injuries	21% with a BSS score = 0-2 had sustained previous netball injuries compared with 37% with a BSS score = 3-4, and 43% with a BSS = 5-9 (p < 0.025). Injuries were common at the ankle (42%), knee (27%), fingers (15%).	JH was associated with increased injuries. A targeted intervention may help reduce injuries in this susceptible group.
Soper K, et al.[17]	Studied 14-26 year old netball players with an JH prevalence of 63% .	Subjects with JH had increased functional test postural instability.	The high JH prevalence was associated with impaired functional movement control.
Stewart D, Burden S.[26]	Studied JH in male first division rugby players to determine if strength protected against joint injury in JH and "tight" players	Overall JH was 24% (12/51). Injury incidence was higher in JH (116.7/1000 hours) than tight (43.6/1000 hours) players (p = 0.034). Group strength differences were not identified.	JH explained group injury rate differences. Groups displayed comparable peak strength.
Sueyoshi T, et al.[79]	Studied JH and ligament injury incidence in high school female volleyball players	Injury group subjects had higher BSS scores than the non-injured group (2.40 ± 1.42, vs. 1.24 ± 1.09, P = .006). Eleven injury group subjects had multiple or recurrent injuries compared to the other 19 individuals in the injury group. For these subjects the mean BSS score = 3.18 ± 1.47 compared to 1.95 ± 1.22 (P = .02).	Female athletes with a high JH score may be more prone to ligament injury and potentially to recurrent ligament injuries.
Tobias JH, et al.[82]	Attempted to determine whether JH was a musculoskeletal pain risk factor among adolescent boys and girls	Musculoskeletal pain at mean age 17.8 years was most common at the lower back (16.1%), shoulder (9.5%), upper back (8.9%), knee (8.8%), neck (8.6%), and ankle/foot (6.8%).	JH was a MS pain risk factor during adolescence, particularly at the shoulder, knee, and ankle/foot. Relationships were strongest with obesity, consistent with a causal pathway where JH creates pain at sites exposed to the greatest forces.
Van Meulenbroek et al.[84]	Studied whether adolescents with asymptomatic JH had lower physical activity levels, muscle strength and performance compared to adolescents without JH .	Adolescents with JH had greater peak knee extensor torque/BW when controlled for age and gender. No other differences were observed.	Adolescents with JH had greater peak knee extensor torque/BW than NH adolescents.
van Rijn RM, et al.[85]	Studied the influence of JH on injury risk among pre-professional contemporary dancers.	The overall mean BSS score was 2.8. The 1-year injury incidence proportion was 67.6% (n = 125), 43.2% (n = 80), and 54.6% (n = 101) for all complaint injuries, substantial injuries, and time-loss injuries, respectively. There was a relationship between having had a previous long lasting injury in the past year and the three injury definitions (p < 0.05).	Dancers were at high risk for JH and injuries, however, these two variables were not associated with each other.
Zhong G, et al.[89]	Attempted to determine if active 3D knee kinematics during treadmill walking of college students with JH was poor compared to college students without	Subjects with JH had greater anterior-posterior AROM (p = 0.026), had greater knee flexion at terminal stance (p = 0.039) and had greater anterior tibial translation during most of the gait cycle (p ≤ 0.05). The JH group had a greater external angle at mid-stance (P = 0.008).	In subjects with JH, poor anterior-posterior translation control might serve an important role in ACL deficiency, knee joint instability, and knee osteoarthritis development.
