# Supplementary material for: Beighton Scoring System Use in Generalized Joint Hypermobility Studies Has Greater Scientific Rigor Than Joint‐Specific or Arthroscopy Joint Hypermobility Studies
Source: Arthrosc Sports Med Rehabil. 2026 May 4;8(2):e70000. doi: 10.1002/ars2.70000 (PMC13307201; doi:10.1002/ars2.70000)
Supplement: Supplementary file 1 — Supplementary Material [file ARS2-8-e70000-s001.zip › ASMAR_SUPPLEMENTAL_TABLE_2Z.pdf]

Supplemental Table 2. Knee joint-specific or arthroscopy JH study publication source, study purpose, results, and conclusion. ACLR = anterior cruciate ligament reconstruction; ALL = anterolateral ligament; ATS = anterior tibial subluxation; ATT = anterior tibial translation; ATTd = anterior tibial translation distance; ATTp = anterior tibial translation peak; ATS = anterior tibial subluxation; BPTB = bone-patellar tendon-bone; FU = follow-up; GR = genu recurvatum; H/Q = hamstring/quadriceps; HR = hazard ratio; HTA = hamstring tendon autograft; IKDC = International Knee Documentation Committee; JH = joint hypermobility; KOOS = Knee Injury and Osteoarthritis Outcome Score; LET = lateral extra-articular tenodesis; LKS = Lysholm Knee Scale; LSI = limb symmetry index; MCID = minimal clinically important difference; MCP = metacarpophalangeal; MRI = magnetic resonance imaging; PROM = patient reported outcome measurement(s); QA = quadriceps autograft; QOL = quality of life; RCT = randomized controlled trial; RTP = return to performance; RTS = return to sports; TASS = Tegner Activity Scale Score; 4S = four strand; 5S = five strand.

Study	Purpose	Results	Conclusion
Astur D, et al.[35]	To evaluate the prevalence of JH and activity level among patients who underwent meniscus and ACL tear surgery.	242 patients had surgery (107 to treat ACL injuries, 75 to treated ACL injuries with meniscus injuries, and 60 to treat meniscus injuries). 45 patients had JH.	JH did not have a negative impact on post-surgical outcomes.
Batty L, et al.[37]	Evaluated the influence of BSS score and GR of 10° representing JH on the relationship between having a high grade preoperative pivot shift test and baseline PROM scores among high re-injury risk patients.	Six factors were associated with a high-grade pivot shift: BSS score (OR = 1.17; 95% CI, 1.06-1.30; p = .002), male gender (OR = 2.30; 95% CI, 1.28-4.13; p = .005), presence of a posterior third medial (OR = 2.55; 95% CI, 1.11-5.84; p = .03) or lateral (OR = 1.76; 95% CI, 1.01-3.08; p = .048) meniscal injury, tibial slope > 9° (OR = 2.35; 95% CI, 1.09-5.07; p = .03), and chronicity > 6 months (OR = 1.7; 95% CI, 1.00-2.88; p = .049). Including GR for JH designation improved BSS score diagnostic utility as a high-grade pivot shift detector. Tibial slope < 9° was associated with pivot only in the presence of a posterior third medial meniscal injury. Patients with a pivot shift had higher baseline 4-Item Pain Intensity Measure scores (mean ± SD, 11 ± 13 vs 8 ± 14; P = .04).	JH, male gender, posterior third medial or lateral meniscal injury, increased posterior tibial slope, and chronicity were associated with a high-grade pivot shift among individuals deemed at high risk for repeat ACL injury. The effect of tibial slope may be accentuated by the presence of meniscal injury, supporting the need for meniscal preservation. Baseline PROMs were similar between patients with and without a high-grade pivot shift.
Brinkman J, et al.[38]	Compared the effects of JH on patient outcomes post-ACLR using an HTA, HTA + LET, or QA	All-soft-tissue QA or the addition of an LET to a HTA both led to decreased graft re-tear rates and less residual ≥ grade 2 pivot shifts compared to HTA graft alone. QA and LET use, however, did not improve PROM at 2-year FU compared to HTA alone. RTS was faster with QA compared to the HTA and HTA+LET; however, the addition of an LET to a HTA did not increase the RTS rate.	High risk patients including those with JH and > grade 2 pivot shifts should undergo ACLR with either a QA or have a LET added to a HTA.
Feller J, et al.[45]	High ACL re-injury risk patients post-primary ACLR with a modified Ellison procedure (25 atients with > 2 of the following risk factors: age < 20 years at surgery, previous contralateral ACLR, family ACL rupture history, JH, grade +3 pivot shift, desire to return to a pivoting sport, or an elite or professional status	The modified Ellison procedure as a LET primary ACLR augmentation produced a low graft rupture rate and was safe in this high re-injury risk cohort.	The procedure showed promise for reducing ACL graft injuries.
Getgood A, et al.[48]	In a multi-center RCT with ≥ 2 years FU, evaluated if adding LET to HA ACLR reduced the clinical failure rate in young patients with an ACL deficient	ACLR with LET reduced clinical failure and graft rupture at 2 years compared with ACLR alone. Pain was less in the ACLR-alone group vs. ACLR + LET at 3 months (p = 0.003). ACLR alone had a greater IKDC and KOOS domain score improvement vs. ACLR +	In a high re-injury risk group of young patients with an ACL-deficient knee, adding a modified Lemaire LET to single-bundle HTA ACLR reduced clinical failure rate at 2 years.

	knee and high re-injury risk: ≥ level 2 competitive pivoting-sport participation, ≥ grade 2 pivot-shift, and JH.	LET at 3 and 6 month FU ($p < 0.05$). At 2-years FU, however, groups did not differ for any PROM, including the Marx Activity Rating Scale ($p > 0.10$).	
Helito C, et al.[51]	Compared functional outcomes, residual instability, and rupture rates in patients with JH undergoing isolated ACLR or combined ACLR and ALL reconstruction.	90 patients undergoing ACLR with JH were evaluated. Mean FU was 29.6 ± 6.2 months for group 1 and 28.1 ± 4.2 months for group 2 (P = .51). Significant group differences were not observed for BSS score, gender, injury duration before ACLR, FU time, preoperative instability, or associated meniscal injuries. The mean age was 29.9 ± 8.1 years in group 1 and 27.0 ± 9.1 years in group 2 (P = .017). At final FU group 2 had better anterior-posterior clinical stability (p = 0.02), better pivot-shift rotatory stability (p = 0.03) and a lower ACLR failure rate (group 1 = 21.7% vs. group 2 = 3.3%, p = .03). Post-surgical IKDC and LKS evaluations did not display group differences.	In patients with JH, combined ACLR and ALL reconstruction resulted in a lower failure rate and improved knee stability compared to isolated ACLR.
Hosseinzadeh N, et al.[52]	Evaluated the influence of JH on patient outcomes following ACLR with a quadruple HTA.	Lachman and pivot shift test results were not different between JH and non-JH patients. Although mean ATT was greater in the H group (7.06 ± 1.41 mm vs. 6.11 ± 1.53 mm in the non-H group, p = 0.006), mean side-to-side differences were not evident (2.25 ± 1.31 mm vs. 2.5 ± 1.44 mm in the control group, p = 0.42). Mean IKDC scores also did not reveal differences between the JH and non-JH groups, respectively (66.1 ± 20.6 vs. 69.9 ± 16.1, p = 0.35). Graft failure occurred in 2 (5.5%) patients of the JH group and 0 in the control group (p = 0.21).	ACLR with quadruple HTA was an adequate treatment for JH patients, at least at short-term FU.
Juul-Kristensen B, et al.[55]	Compared knee function in children and adults with or without JH, a minimum of one JH knee, no knee pain in children and adults.	Adults with JH had lower knee function (KOOS: pain, p = 0.001; symptoms p = 0.001; ADL p = 0.001; Sport/Recreation p = 0.003; knee-related QOL p < 0.001) scores, and lower H/Q strength ratio levels (0.46 vs. 0.54, p = 0.046) compared to adults with no H regardless of age and knee pain level. Both groups had normal physical fitness, isokinetic knee strength, and H/Q ratio (only children were evaluated for this).	Children at 10 years of age with JH had normal function, however, adults with JH had impaired knee function. They suggested that to track knee functional impairment development risk, children with JH should be followed longitudinally and greater attention to knee function should be given individuals with JH whose parents have the same condition.
Keizer M, et al.[57]	Hypothesized that patients post-ACLR compensated for anterior tibial translation distance (ATTd) by developing neuromuscular activation strategies which would be more effective in copers than in non-copers.	There was no relationship between peak ATT and ATTd in copers; however, there was a positive correlation between ATTp and ATTd in the surgical knee of non-copers. There was a positive correlation between BSS score and ATTp and between BSS score and ATTd in both copers and noncopers at the surgical knee. Copers had a negative correlation between surgical side ATTd and gastrocnemius neuromuscular activity during landing. Noncopers had a positive correlation between surgical knee ATTd and knee flexion moment during single leg hop landing.	Copers used different landing techniques than noncopers. Patients who returned to sports after ACLR had sufficient plantar flexor neuromuscular activation to limit ATTd.
Kim S, et al.[59]	Compared 2- and 5-year outcomes post-ACLR between patients with and without JH and compared two different graft types used for ACLR in patients with JH.	At 2 -years post-ACLR patients with JH who had received HTA had poorer outcomes than those without JH. Irrespective of graft type, at 5-year FU patients with JH had poorer outcomes than patients without JH. Comparison by graft type revealed that in patients with JH a BPTB graft provided better stability and functional outcomes than HTA at both	Less satisfactory stability and functional outcomes were noted in patients with JH compared to patients without JH and the BPTB graft achieved better results than the HTA.

		2- and 5-year FU. Outcome comparisons at 2 and 5 years demonstrated that stability and functional outcomes were more likely to deteriorate in patients with JH. In patients with JH the BPTB graft provided better stability and functional outcomes than HTA at both 2- and 5-year FU.	
Larson C, et al.[62]	Determined if general JH and contralateral GR affected ACLR failure rates.	41/183 consecutive patients were categorized as JH. At mean 6 year FU. IKDC (p = .003), Cincinnati (p = .001), and LKS scores (p < .001) were better in the non-JH group for patients with an intact graft. The failure rate was higher in the JH group (10 knees, 24.4% failure rate) compared with the non-JH group (11 knees, 7.7% failure rate) (p = .006). The overall ACL injury rate (ACL graft injury, excessive graft laxity, plus contralateral ACL tear) was higher in the JH group (34.1%) compared with the non-JH group (12.0%) (p = .002). Heel height > 5 cm (p = .009) and fifth MCP extension > 90° (p = .006) were independently predictive of failure for the entire study population.	Higher ACLR graft failure rates and lower subjective outcome scores were observed in patients with JH. Heel height and fifth MCP hyperextension were most predictive of ACL injury/reinjury and poorer outcomes. Nearly 1/3rd of patients with JH sustained a contralateral ACL tear, ipsilateral graft failure, or had excessive graft laxity.
Lindskog J, et al.[64]	Compared RTS (TASS ≥ 6) and RTP among patients with or without JH post-ACLR	Fewer patients with JH achieved RTS compared with patients without H (49.2% vs. 57.3%, OR: 0.720, p = 0.041). Patients with JH were less symmetrical (LSI) for knee extensor strength at the time of RTP compared with patients without JH (87.3% ± 13.5 vs. 91.7% ± 14.3, Cohen's d = 0.142, p = 0.022).	Other than for RTP, no other differences were found between groups regarding any muscle function tests or PROM.
Lodhia, P. et al.[65]	Investigated the clinical outcome differences between 4-strand (4S) and 5-strand (5S) HTA for ACLR in patients who underwent ACLR alone or concomitantly with an LET procedure.	Of the 618 patients randomized in the study, 399 (228 male; 57%) fit the inclusion criteria. Of these, 191 and 208 patients underwent 4S and 5S configurations of HTA ACLR, respectively, with a minimum 8-mm graft diameter. There were no group differences for primary outcomes of rotatory knee stability, graft failure, Lachmans or Pivot shift test results at 24 month FU. Secondary outcomes revealed no differences for ACL-QoL and IKDC scores.	At 24-month FU there were no differences in clinical failure rates and PROM in patients with ≥ 8-mm diameter 4S and 5S HTA for ACLR or ACLR + LET. The 5S HTA was deemed a viable large-diameter ACL graft option.
Marmura H, et al.[67]	Studied KOOS validity for determining perceived function among young, active patients "at risk" for re-injury post-ACLR based on ≥ 2 of the following factors: pivoting sport participation, ≥ + 2 grade pivot shift, and JH or GR > 10°.	The KOOS five-factor structure had poor structural validity for this population.	Recommended that a modified KOOS was needed to better reflect and interpret the outcomes and recovery trajectory in this high-functioning patient population
Parmar R, et al.[71]	Evaluated the effectiveness of adding LET to ACLR in competitive female soccer athletes with greater pre-operative JH.	133 players who underwent ACLR met inclusion criteria, including 43 that received an ACLR + LET and 90 patients who underwent isolated ACLR. Average FU was 39.0 and 36.1 months in the LET group and the control group, respectively. Patients who underwent LET more frequently had JH (48.8% vs 18.9%; p < .001) and had higher median BSS scores (3.0 vs 1.0; p < .001) than those without LET. There was similar graft failure rates between patients who underwent LET compared with the comparison group (4.7% vs 3.0%; p = .658). On the basis of MCID thresholds, there were no clinically relevant differences in IKDC or LKS scores between the 2 groups. Comparable RTS rates were also	The addition of LET during ACLR in female soccer players with preoperative JH yielded graft re-tear and RTS rates comparable with those of athletes without JH.

		observed between the LET and comparison cohorts (90.7% vs 85.6%; p = .807).	
Pfeiffer TR, et al.[72]	Tested 98 college athletes median 20 years of age (range = 18-25) with no knee injury history for bilateral ATT translation, JH and GR in addition to IKDC and Marx activity scores. A standardized pivot shift test was also performed at both knees and quantified using image analysis technology.	The average lateral compartment ATT during the pivot shift test was 1.6-mm (range = 0.1 - 7.1-mm) with a mean side-to-side difference of 0.6-mm (range = 0 - 2.7 mm). The average Lachman test ATT was 9.0-mm (range = 2 -15-mm). Lateral compartment pivot shift ATT was higher in females (median = 1.6-mm; range = 0.3 - 4.9-mm) than in males (median = 1.1-mm, range = 0.1 - 7.1-mm) (p < 0.05). Lateral compartment pivot shift ATT significantly correlated with Lachman test ATT (r = 0.34; p < 0.05).	There was no significant correlation between lateral compartment ATT during the pivot shift test, JH and GR. Female gender was associated with increased rotatory knee laxity measured during the pivot shift test and anterior translation during the Lachman test.
Sahin S, et al.[75]	Evaluated the relationship between JH and knee biomechanical risk factors among adolescent volleyball players.	Peak knee valgus during the drop landing vertical jump-landing (r = 0.487, p = 0.014), single leg drop landing (r = 0.478, p = 0.016), and single leg squat (r = 0.439, p = 0.028) tasks were moderately correlated to JH.	Adolescent volleyball players with higher BSS scores and greater peak knee valgus could benefit from a targeted neuromuscular training or injury prevention program.
Sundemo D, et al.[80]	Studied whether greater rotatory knee laxity was associated with greater JH in ACL-injured and contralateral knees.	96 patients had complete datasets, 83 and 13 were in the low and high JH groups, respectively. In anesthetized patients, there was a significant relationship between JH and quantitative pivot shift (r = 0.235, p < 0.05). When analyzing the same knee, multivariate analysis adjusted for meniscal injury, age and gender revealed an increased odds ratio for patients with increased lateral compartment translation to be part of the high JH (OR = 1.86, 95% CI 1.10-3.17, p < 0.05).	A weak relationship was observed between JH and the contralateral healthy knee, indicating increased rotatory knee laxity. JH did not correlate with rotatory knee laxity in ACL-injured knees.
Sundemo D, et al.[81]	Evaluated whether JH influenced post-ACLR results, including RTS (PROM, hop tests, muscular strength, and ACL re-injury, in patients 1 year post-ACLR.	356 patients (41% males) were included, of which 76 (24% male) were categorized as having JH. Patients with JH had an inferior pre-surgery LSI for knee extensor strength LSI (mean 81.6% ± 16.4 vs. 91.4% ± 15.9, p = 0.02) and knee flexor strength LSI (mean 91.9% vs. 99.1%, p = 0.047) compared to patients without JH. There were no group differences for the primary outcome, nor for any of the other post-operative outcomes. Nine patients (11.8%) in the JH group experienced ACL re-injury, compared with 13 patients (4.6%) in the control group (n.s.).	At 1 year post-ACLR JH did not affect post-operative patient satisfaction, strength or functional outcome. No conclusive statements could be made regarding the influence of JH on ACL re-injury risk.
Vaishya R & Hasija R[83]	Compared JH rates in patients with and without ACL injury.	127 (60.5%) of the patients with ACL injury and 23 (25.5%) of the controls had JH (p < 0.01). Among them, 58% and 24% were men and 65% and 29% were women, respectively. Female gender was associated with JH. Patients with ACL injury were more likely to have JH with an OR = 4.46.	JH was more common in patients with ACL injury.
Westin M, et al.[86]	Studied the possible intrinsic risk factors for ACLR re-injury in competitive alpine skiers from a Swedish high school.	Twelve of 31 students (39%), 10 female and 2 male skiers, aged 16.5 (SD = 0.5) years, sustained ACLR re-injury. Additionally, 10/12 ACLR re-injuries occurred at 10-23 months after the first injury (14.8 months ± 4.7) and 2 ACLR re-injuries occurred at 29 and 47 months, respectively, from the first injury. 8 ACLR re-injuries were at the ipsilateral knee and 4 at the contralateral knee. There were no group differences with respect to LE muscle flexibility, JH, and one leg hop for distance or square hop tests. Side-to-side differences were found with respect to knee joint laxity, >3 mm, (p = 0.02), and the side	Side-to-side differences in the side hop test and anterior translational knee joint laxity may predispose ACLR re-injury in competitive adolescent alpine skiers.

		hop test (p = 0.04). Short Form 36 was not predictive of ACL re-injury.	
Zhang ZY, et al.[87]	To better discern ACL graft failure risk in patients with ACL graft failure compared with primary ACL tear patients.	109 primary ACLR patients (mean age = 33.7 years, range 15-71), and 90 revision ACLR patients (mean age = 32.9 years, range 16-65) participated. The revision ACLR group had greater JH and greater side-to-side quadricep circumference differences (2-cm vs. 0- cm; p < .001) compared with the primary ACLR group. Family history of ACL tear was more likely in the revision group (47.8% vs. 16.5%; p < .001). The revision group also had greater lateral posterior tibial slopes (7.9° vs. 6.2°), anterolateral tibial subluxation (7.1 vs. 4.9 mm), and anteromedial tibia subluxation (2.7-mm vs. 0.5-mm; all p < .005). In the revision group, 48 cases (66.7%) had femoral tunnel malposition in the deep-shallow position and in 24 cases (33.3%) femoral tunnel malposition was in the high-low position. In 7 cases (9.7%) the tibial tunnel was malpositioned in the medial-lateral plane and in 39 cases (54.2%) the tibial tunnel was malpositioned in the anterior-posterior plane. 56 patients (77.8%) had tunnel malposition in ≥ 2 positions. Allograft tissue was used for the index ACLR in 28% in the revision group vs. 14.7% in the primary group.	JH, quadriceps circumference side-to-side difference, family ACL tear history, lateral posterior tibial slope, anterolateral tibial subluxation, and anteromedial tibia subluxation differed between the primary and revision ACLR groups. There was also a higher tunnel malposition rate in the revision ACLR group.
Ziegler C, et al.[89]	Using MRI this study attempted to quantitatively describe pre-surgical ATS and RTS in ACL-injured and ACL-intact knees and identify related factors for ATS and RTS	Increased lateral ATS (p < 0.0001), medial ATS (p < 0.0001) and RTS (P = 0.0479) were observed in ACL-injured knees compared with the control group. Increased PTS, JH, meniscal injury and long injury-to-MRI time were related to increased ATS. Factors related to an increased RTS were increased lateral PTS, JH, lateral meniscal injury, and left side.	In ACL-injured knees the tibia not only subluxated anteriorly in both lateral and medial compartments, but also rotated internally. During pre-surgical planning, attention should be paid to factors related to altered tibiofemoral position.
Zsidai B, et al.[9]	Studied the 12-month risk of a second ACL injury in patients with and without JH who RTS at competition level post-ACLR.	Demographics, outcome data and second ACL injury incidence within 12 months of RTS, defined as a new ipsilateral or contralateral ACL, were compared between patients with and without JH. Within 12 months of RTS, 7 (14.0%) patients with JH and 5 (2.9%) without JH had a second ACL injury (p = 0.012). The odds of sustaining a second ipsilateral or contralateral ACL injury were 5.53 (95% CI 1.67-18.29) higher in patients with JH compared with patients without JH (p = 0.014). The lifetime HR for a second ACL injury post-RTS was 4.24 (95% CI 2.05-8.80; p=0.0001) in patients with JH. No between-group differences were observed for PROM.	Patients with JH who undergo ACLR had a > 5X greater OR for sustaining a second ACL injury post-RTS. The importance of H assessment should be emphasized in patients who desire to return to high-intensity sports post-ACLR.
